# Disease-related blood-based differential methylation in cystic fibrosis and its representation in lung cancer revealed a regulatory locus in *PKP3* in lung epithelial cells

**DOI:** 10.1080/15592294.2021.1959976

**Published:** 2021-08-20

**Authors:** Esther Schamschula, Angelika Lahnsteiner, Yassen Assenov, Wolfgang Hagmann, Nadja Zaborsky, Markus Wiederstein, Anna Strobl, Frauke Stanke, Thomas Muley, Christoph Plass, Burkhard Tümmler, Angela Risch

**Affiliations:** a Department of Biosciences, University of Salzburg, Salzburg, Austria; b Division of Cancer Epigenomics, German Cancer Research Center (DKFZ), Heidelberg, Germany; c Department of Internal Medicine III with Haematology, Medical Oncology, Haemostaseology, Infectiology and Rheumatology, Oncologic Center, Salzburg Cancer Research Institute - Laboratory for Immunological and Molecular Cancer Research (SCRI-LIMCR), Paracelsus Medical University, Salzburg, Austria; d Cancer Cluster Salzburg, Salzburg, Austria; e Clinical Research Group, Clinic for Pediatric Pneumology, Allergology and Neonatology, Hannover, Germany; f Biomedical Research in Endstage and Obstructive Lung Disease Hannover (BREATH), German Center for Lung Research, Hannover Medical School, Hannover, Germany; g Translational Research Unit, Thoraxklinik Heidelberg, University of Heidelberg, Germany; h Translational Lung Research Center Heidelberg (TLRC-H), Member of the German Center for Lung Research (DZL), Heidelberg, Germany

**Keywords:** Cystic fibrosis, lung cancer, lung inflammation, monozygotic twins, DNA methylation

## Abstract

Cystic fibrosis (CF) is a monogenic disease, characterized by massive chronic lung inflammation. The observed variability in clinical phenotypes in monozygotic CF twins is likely associated with the extent of inflammation. This study sought to investigate inflammation-related aberrant DNA methylation in CF twins and to determine to what extent acquired methylation changes may be associated with lung cancer.

Blood-based genome-wide DNA methylation analysis was performed to compare the DNA methylomes of monozygotic twins, from the European CF Twin and Sibling Study with various degrees of disease severity. Putatively inflammation-related and differentially methylated positions were selected from a large lung cancer case-control study and investigated in blood by targeted bisulphite next-generation-sequencing. An inflammation-related locus located in the *Plakophilin-3* (*PKP3*) gene was functionally analysed regarding promoter and enhancer activity in presence and absence of methylation using luciferase reporter assays.

We confirmed in a unique cohort that monozygotic twins, even if clinically discordant, have only minor differences in global DNA methylation patterns and blood cell composition. Further, we determined the most differentially methylated positions, a high proportion of which are blood cell-type-specific, whereas others may be acquired and thus have potential relevance in the context of inflammation as lung cancer risk factors. We identified a sequence in the gene body of *PKP3* which is hypermethylated in blood from CF twins with severe phenotype and highly variably methylated in lung cancer patients and controls, independent of known clinical parameters, and showed that this region exhibits methylation-dependent promoter activity in lung epithelial cells.

## Introduction

Cystic fibrosis (CF) is an autosomal recessive monogenic disease caused by mutations in the cystic fibrosis transmembrane conductance regulator (*CFTR*) gene which is located on chromosome 7q31.2 and was discovered in 1989 [1]. It affects approximately over 1:2000 new-borns in the Caucasian population [2].

More than 2000 different genetic variants in *CFTR*, including missense, frameshift, splicing and nonsense mutations as well as in-frame deletions and insertions, are known to be causative for various CF phenotypes [3]. CFTR protein deficiency causes excessive inflammatory processes in the affected organs, mainly the lung, pancreas and intestines [4,5]. Major contributors to the severity of this condition are respiratory malfunctions and malnutrition. These symptoms are reflected by the clinical parameters forced expiratory volume in 1 second percent (FEV1%) and weight expected for height percent (wfh%), both referring to the non-CF population and representative for pulmonary and nutritional status, respectively, which together are considered to define the disease severity and subsequently intra-pair discordance [6]. Both parameters are associated with the chronic inflammation of the affected organs [7,8].

Considering that CF is a monogenic disease, the severity of the lung condition is remarkably variable in different individuals. The most evident cause for this phenomenon is the high number of distinct *CFTR* mutation types, leading to different CFTR protein defects and impaired functions [3]. Additionally, a number of genetic modifiers affecting the CF phenotype have been identified in various genetic association studies and have been shown to account for more than 50% of variation in the lung disease phenotype [9–12]. Interestingly, clinical discordance has been observed even in monozygotic twins with CF. This implies a role of environment and epigenetics in determining disease severity in addition to the genetic factors [13,14]. Phenotypical discordance in monozygotic twins is plausibly associated with epigenetic effects, as the respective twins are genetically identical and typically share early-life environmental exposure. Due to the reduced confounders and cohort effects in twin studies, even small sample groups are appropriate for identification of phenotype-associated epigenetic markers and mechanisms [15,16].

Several twin studies have been successfully performed to investigate epigenetic patterns, such as DNA methylation, in the context of lung function, inflammatory diseases and cancer [13,17–21]. Differentially methylated loci associated with diseases such as cancer are considered promising as biomarkers for risk, development and progression of diseases [20,22,23, 24]. DNA methylation patterns, although cell-type and tissue specific can be reflected in clinically accessible surrogate tissues, such as whole blood [25–29]. In concert with other epigenetic mechanisms, DNA methylation decreases the accessibility of genomic regions and therefore has physiological functional relevance for genomic stability, chromatin structure, imprinting, transcription factor binding, splicing, silencing of retro-transposal, repetitive elements and gene expression (e.g., oncogenes) as well as for activation of gene expression (e.g., tumour suppressor genes) and transcription of antisense transcripts [30–33].

Consequently, aberrant DNA methylation or demethylation is also functionally associated with a variety of diseases, among them inflammatory diseases and cancer [31,34–37].

It has been shown that chronic inflammation in the lung and other tissues alters DNA methylation patterns via distinct mechanisms involving inflammatory mediators and infectious agents [38–41]. Chronic inflammation and infections are strongly connected with cancer risk, initiation and progression and have been described as causative and consequential events in cancer development [42,43].

On the one hand, infectious agents generate metabolites, on the other hand, the host´s immune system produces cytokines, chemokines, growth factors and free radicals. If unresolved, chronic inflammation causes a constant disordered environment, finally leading to genetic and epigenetic aberrations promoting neoplasia [44,45]. Hence, infection and inflammation can both increase cancer risk via multiple mechanisms of which many have yet to be clarified. A better understanding of these mechanisms is essential in order to improve cancer intervention strategies.

Altered DNA methylation is one mechanism promoting cancer development in an inflammatory environment, as has been shown for infections with the Human Papilloma virus, *Helicobacter pylori*, Epstein–Barr Virus and Hepatitis B and C viruses [46, 47–52]. Chronic inflammatory lung diseases such as chronic obstructive pulmonary disease (COPD) are associated with an increased risk for lung cancer [53, 54, 55]. Furthermore, chronic inflammation of the lung has been shown to be implicated in lung cancer development also via epigenetic mechanisms [56,57].

Although CF has not been demonstrated to be associated with lung cancer risk, we consider CF an adequate model for investigating the impact of massive chronic inflammation of the lung and its implications in other inflammation-related lung diseases such as lung cancer [58]. It has been shown that the well-known modifier of CF lung disease, *Mucin 4* (*MUC4*) and *Mucin 5AC* (*MUC5AC*) are associated with lung cancer risk and prognosis, respectively [11,59,60]. Further CF lung modifier genes, such as *ETS homologous factor* (*EHF*), (*Epithelial splicing regulatory protein 2*) *ESRP2* and *macrophage migration inhibitory factor* (*MIF*) have functional roles in epithelial to mesenchymal transition, a process that is also epigenetically regulated and plays a major role in lung cancer development [61–65].

*Glutathione S-transferase M 1* (*GSTM1*), involved in detoxification pathways, is related to lung cancer risk in an environment-dependent manner and modifies lung disease severity in CF as well [11,66,67]. *Transforming growth factor beta 1* (*TGFβ1*), associated with inflammation and lung cancer risk and the smoking-related differentially methylated gene *Aryl-hydrocarbon receptor repressor* (*AHRR*) are likewise modifiers of CF lung disease [11,65,68,69].

In the present study we aim to identify novel mechanisms that may play a role in inflammation-related lung carcinogenesis. We investigated aberrant DNA methylation patterns putatively acquired by chronic inflammation of the lung, their appearance in the lung cancer context and their functional implications in lung diseases, particularly lung cancer. For this purpose, we examined blood-based genome-wide DNA methylation patterns in monozygotic CF twin pairs with distinct phenotypical discordance from the European CF Twin and Sibling Study [6]. In order to study inflammation-related epigenetic alterations, genome-wide DNA methylation data were obtained for 22 monozygotic twin pairs with CF. The methylomes were characterized with respect to the clinical parameters. Discordance-dependent differentially methylated loci were identified by an unbiased selection process. Differentially methylated loci, potentially interesting in the context of chronic lung inflammation and lung cancer, were tested in whole blood samples of a large lung cancer case-control cohort to discover epigenetic alterations with possible functional implications in inflammation-related lung cancer. For the most interesting locus, functional analysis has been conducted in lung epithelial cells.

## Material and methods

### Subjects and samples

Illumina 450 K methylation analysis was conducted for 22 monozygotic twin pairs with Cystic Fibrosis from the **European CF Twin and Sibling Study**. Spirometry-based lung function measurements were available and the clinical severity (DfO: distance from origin) and the phenotypical intra-pair discordance (Delta) had been calculated based on lung function, reflected by FEV1% (forced expiratory volume in 1 second percent) and wfh% (weight expected for height percent), representative for nutritional status as described previously [6]. With respect to their intra-pair discordance, the twin pairs were assigned to the categories concordant (n = 6), intermediate (n = 6), discordant (n = 6) and NA (unknown discordance) (n = 4) (Table 1).

**Table 1.** Clinical characteristics of the CF twins.

		Twin pair category				
		concordant	intermediate	discordant	unknown discordance	Σ
**clinical parameter**		(n = 12 individuals)	(n = 12 individuals)	(n = 12 individuals)	(n = 8 individuals)	(n = 44 individuals)
**age [y]**		19.3 ± 9.6	14.6 ± 7.3	17.0 ± 10.2	3.0 ± 1.2	14.4 ± 10.1
**weight [kg]**					11.3 ± 3.6	
**height [cm]**					86.5 ± 14.6	
**DfO**		562.3 ± 235.8	466.8 ± 133.6	466.9 ± 203.4		
**Delta**		80.5 ± 36.7	164.0 ± 31.5	366.2 ± 36.5		
**female/male ratio**		2.0	0.2	2.0	3.0	1.2
***CFTR* genotype**	F508d/F580d	6	6	8	4	24
	F508d/D579G	2				2
	F508d/1525-2A-G	2				2
	F508d/3849 + 10kbC-T	2				2
	3601–2A-G/3601-2A-G		2			2
	F508d/2183 AA-G		2			2
	Q39X/Q552X		2			2
	2183AA-G/IVS8:TG15-5 T			2		2
	F508d/G542X			2		2
	F508d/4016insT				2	2
	F508d/1717-1 G-A				2	2

CF: cystic fibrosis; NA: unknown discordance; DfO: distance from origin, corresponds to severity; Delta: intra-pair discordance; *CFTR*: cystic fibrosis transmembrane conductance regulator

For targeted bisulphite sequencing-based methylation analysis 109 lung cancer patients and 109 healthy controls from the **HD lung cancer case–control cohort** [70,71] were pair-wise matched by sex, age (± 0 years), packyears (±0 years) and smoking status (current, ex and never smokers), in a nested case-control study design (Table 2).

**Table 2.** Clinical characteristics of lung cancer cases and healthy controls individually matched by age, gender, smoking status and packyears.

variables		cases (n = 109)	controls (n = 109)
**sex**	**female**	**50**	**50**
	**male**	**59**	**59**
**mean age [y]**		**60.6 ± 10.2**	**60.6 ± 10.2**
**mean packyears [y]**		**15.2 ± 24.3**	**15.2 ± 24.3**
**smoking status**	**current**	**25**	**25**
	**ex**	**17**	**17**
	**never**	**67**	**67**
**cancer type**	**no cancer**	**0**	**109**
	**SCC**	**18**	**0**
	**LCC**	**8**	**0**
	**adenocarcinoma**	**59**	**0**
	**carcinoid**	**6**	**0**
	**NSCLC**	**2**	**0**
	**SCLC**	**14**	**0**
	**mixed**	**2**	**0**

n: number of samples; SCC: squamous cell carcinoma; LCC: large cell lung cancer; NSCLC: non-small cell lung cancer; SCLC: small cell lung cancer .

### Epigenome-wide DNA methylation analysis

From all subjects, frozen peripheral blood samples were used to extract genomic DNA (gDNA). The gDNA was bisulphite-converted with the EZ DNA Methylation™ Kit (Zymo Research). DNA methylation was quantified with the Illumina 450 K BeadChip Array (Illumina). Bioinformatical analysis and quality control of 450 K methylation data were performed with the R package RnBeads [72].

### iDMP selection process

For selection of disease-related differentially methylated positions, probes with missing values were removed. Further, positions located in repetitive sequence according to Price et al. (n_bp_repetitive ≥1) were ignored [73]. Intra-pair differential methylation was calculated as DM = β_severe_ – β_mild_, where β is the methylation value corresponding to % methylation of the more or less severely affected twin.

For discordance-dependent differentially methylated probes the following assumptions were made: (1) they are strongly differentially methylated, arbitrarily defined as DM±≥0.1 (10%) only in discordant twin pairs and twin pairs with unknown discordance, (2) they are not differentially methylated, defined as DM≤±0.05 (5%) in concordant twin pairs. Methylation differences below 5% at single CpG nucleotides may not be reliably detectable with different methods, such as pyrosequencing [74].

Probes were considered one-directionally differentially methylated if (1) the correlation between methylation values and individual severity (DfO), (2) the correlation between methylation differences and intra-pair discordance (Delta) and (3) the mean methylation differences ($\bar{DM}$) consistently indicate a hyper- or hypo-methylation (i.e., positive DfO value, negative Delta value and negative $\bar{DM}$ value or *vice versa*).

Correlations were calculated according to:

$$CoR=\frac{\sum^{(}x_{i}-\bar{x)}(y_{i}-\bar{y)}}{\sqrt{\sum^{(}x_{i}-{\bar{x)}}^{2}(y_{i}-{\bar{y)}}^{2}}}$$

where x is the severity or discordance matrix, y the methylation value or methylation difference matrix and i is one severity or discordance value corresponding to one methylation value or methylation difference, respectively.

Identification of positions significantly differentially methylated between different blood-cell-types, was done with an F-test (described in statistical analyses), using a publicly available dataset. Briefly, Reinius et al. sorted and performed cell-type specific genome-wide DNA methylation analysis with different blood cell-types obtained from six healthy individuals [75]. Calculations in our study are based on granulocytes, monocytes, CD4^+^ and CD8^+^ T cells, B cells and natural killer cells.

### TCGA data analysis

The cancer genome atlas (TCGA) research network DNA methylation data (https://www.cancer.gov/tcga) obtained with the Illumina 450K BeadChip, were downloaded from the TCGA Wanderer for lung adenocarcinoma (LUAD) and lung squamous cell carcinoma (LUSC) and corresponding normal adjacent tissues [76]. Probes with a methylation difference of ≥5% between LUAD (32 normal samples, 463 tumour samples) and LUSC (43 normal samples, 361 tumour samples) and normal lung tissue, in the same direction as in the CF twin cohort, were considered to be differentially methylated.

### ENCODE data analysis

Chromatin immunoprecipitation DNA-sequencing (ChIP-seq) data from nine different cell lines (NHLF, NHEK, K562, HUVEC, HSMM, HMEC, HepG2, H1-hESC and GM12878) from the Broad institute were considered using the UCSC genome browser [77–80].

### Assay design for targeted bisulphite next-generation sequencing (NGS)

Twenty-one targeted assays, corresponding to 20 genomic regions were designed using the PyroMark Assay Design Software 2.0. Primer sequences are provided in Supplementary Table 1. The assays were designed based on the UCSC Genome Browser Human Feb. 2009 (GRCh37/hg19) Assembly (http://genome.ucsc.edu/cgi-bin/hgGateway?db=hg19).

### Targeted bisulphite next-generation sequencing

Whole blood was obtained from the study subjects and gDNA was isolated. Further processing involved multiple kits which were always used according to the manufacturer’s standard instructions, with exceptions detailed below. Concentrations were determined with the Qubit 2.0. (Thermo Fisher Scientific) using the Qubit dsDNA Broad Range Assay (Thermo Fisher Scientific). Five hundred ng gDNA from each donor was bisulphite-converted with the EZ DNA Methylation™ Kit (Zymo Research) using the alternative incubation conditions for the Illumina Infinium® Methylation assay and eluted in 2 × 30 µl instead of 1 × 10 µl. For the amplification of the target regions, the HotStarTaq DNA polymerase (Qiagen) was used. Ten multiplex PCR reactions were performed for 21 regions as shown in Supplementary Table 2. The amplification products were cleaned up with AMPureXP Beads (Agencourt) to remove primer dimers in a sample:beads ratio of 1:0.8 prior to determination of concentration with the Qubit dsDNA High Sensitivity Kit (Thermo Fisher Scientific). Amplicons were pooled so that each amplicon was represented in an equimolar amount within a given sample. Eight cycles of index PCRs were performed with the NexteraXT Index Kit v2 Set A and B (Illumina) at 55°C annealing temperature and 30 sec elongation time using the KAPA HiFi HotStart Mix (Roche). The resulting indexed amplicons were purified with AMPureXP Beads (Agencourt) to remove primer dimers in a sample:beads ratio of 1:0.8 prior to determination of concentration with the Qubit dsDNA Broad Range Kit (Thermo Fisher Scientific). All samples were equimolarly pooled and again cleaned up with AMPureXP Beads (Agencourt) to remove primer dimers in a sample:beads ratio of 1:0.8 prior to determination of concentration with the Qubit dsDNA Broad Range Kit (Thermo Fisher Scientific). The quality and size of the library was verified using the Fragment Analyser Standard Sensitivity NGS Fragment 1–6000bp Kit (Agilent) on the Fragment Analyser (Agilent). The final library was denatured and diluted according to the MiSeq System Denature and Dilute Libraries Guide (Illumina) and library concentration was determined with the Qubit dsDNA High Sensitivity Kit (Thermo Fisher Scientific). The final libraries were spiked with 25% PhiX Control v3 (Illumina), diluted to 8pM and 300bp paired-end sequenced on a MiSeqDx instrument (Illumina) with the MiSeq Reagent Kit V3 600 cycles (Illumina). Samples from cases and controls were sequenced on the same flow cell.

### Bioinformatical analyses of targeted bisulphite NGS data

Demultiplexed FASTQ files from two runs on the MiSeq (Illumina) were unzipped and merged sample-wise. Prior to alignment, reads were **quality trimmed** with Trim Galore! (Babraham Bioinformatics), (https://github.com/FelixKrueger/TrimGalore) using Cutadapt version 1.12 for paired-end reads. The quality encoding type was set ASCII+33 (phred 33). Low-quality ends were removed with a threshold of phred score 20. The Nextera Transposase was assumed as adaptor sequence. The maximum trimming error rate was set to 0.1, the minimum required adapter overlap (stringency) was specified as 2 bp and the minimum required sequence length for both reads, before a sequence pair gets removed, was set to 20 bp. Trimmed reads were aligned with the **methylation analysis pipeline** ‘bicycle’ in the paired-end mode with a maximum valid read length of 1000 and phred33 as quality settings for bowtie2 [81]. The UCSC Genome Browser on Human Feb. 2009 (GRCh37/hg19) Assembly was used (http://hgdownload.soe.ucsc.edu/goldenPath/hg19/bigZips/hg19.2bit) as reference genome. The obtained file was converted to a FASTA file, which can be used by bicycle with twoBitToFa (http://hgdownload.soe.ucsc.edu/admin/exe/linux.x86_64/twoBitToFa). Methylation analysis with ‘bicycle’ was performed with a fixed error rate computation mode (Watson error rate = 0.01, Crick error rate = 0.01). Non-correctly bisulphite-converted reads, reads aligned to both Watson (+) and Crick (-) strands and reads with more than one possible alignment were ignored. Mean coverage represents the mean read depth across all cytosines in a region.

### Statistical analyses

Calculation of **95% confidence interval** was performed with the CI function from the ‘Rmisc’ R-package. All statistical analyses were performed with R and RStudio software if not stated differently.

**The cell-type contribution** was estimated with the R package RnBeads applying the algorithm by Houseman et al. [72,82]. As a reference, data from Reinius et al. were used [75].

An **F-test** was done with GEO2R (https://www.ncbi.nlm.nih.gov/geo/geo2r/) using the dataset of Reinius et al. to identify cell-type specific differentially methylated positions. For this purpose, the methylation values of granulocytes, CD4^+^ T cells, CD 8^+^ T cells, CD14^+^ monocytes, CD19^+^ B cells and CD56^+^ natural killer cells were taken into account [75]. P-values were false discovery rate (FDR) corrected (Bonferroni) and values below 0.05 were considered statistically significant.

The non-parametric **Wilcoxon rank sum test** was performed one-sided and unpaired with a confidence interval of 0.95. The exact p-value was computed and then rounded to the second digit. The function wilcox.test was used.

**Enrichment analysis** was executed with one-sided Fisher´s exact test using the fisher.test function. Gene association, CGI relation and regulatory region were characterized according to the Illumina HumanMethylation450 v1.2 Manifest file. Gene association corresponds to functional regions with UCSC genes that a probe is associated with. CGI relation is equivalent to the location of the CpG relative to the CpG island. Enhancers were defined as predicted enhancer elements, DHS as experimentally determined DNase I Hypersensitivity Sites which correspond to open chromatin (ENCODE project). Promoters and cell-type specific promoters are promoter-associated and cell-type-dependent promoter-associated probes, respectively, provided by the Methylation Consortium. Telomeric and subtelomeric regions were defined as the distal 500 kb of each chromosome arm [83]. Imprinted regions were specified according to the Illumina HumanMethylation450 BeadChip (v1.2, extended annotation) file. Probes that overlap with the stretch of 1500 bp around the transcription start site of any of the transcripts of known imprinted genes were defined as imprinted.

**The power-analysis** was done with the power.t.test function using standard deviations of the candidate positions in the whole blood of 796 healthy individuals from case-control pairs from the NOWAC, MCCS, NSHDS and EPIC HD studies [22] **(Supplementary Table 3)**. The power to detect methylation differences ≥5% with a two-sided paired Student´s t-test with a number of 109 observations per group and a Type I error probability of 5% was calculated **(Supplementary Table 4)**.

The **paired Student´s t-test** was performed two-sided with a confidence interval of 0.95 with the function t.test. The two variances have been treated as being equal and NA values were omitted. P-values were corrected for false discovery rate with the R package q-value.

The **principal component analysis** was executed with the methylation values obtained from 218 samples using the default settings of the prcomp function from the stats package.

The **Pearson correlation** between numerical clinical parameters (age and packyears) and mean methylation values per region was calculated using the cor function. The NAs were removed.

The **Pearson correlation** between the global mean methylation difference and discordance (Delta) and age was calculated using the cor function. The NAs were removed.

The **coefficient of determination**, corresponding to $\frac{\%\;\;of\;\;variance}{100}$ in the mean methylation per region explained by the respective nominal parameters (sex, cancer histology, cancer occurrence, and smoking status) was calculated by performing a multiple regression using the lm function. The NAs were removed.

**Gene-ontology enrichment analysis** was performed for genes with inflammation-related differentially methylated positions in their gene body or promoter for the terms biological process, molecular function and cellular component with a Fisher´s Exact test, FDR was calculated.

**Welch Two Sample** t-test was performed with the relative luciferase activity fold-change values. Luciferase activity values were normalized for firefly activity. The fold-change was calculated and compared to the empty vector as a reference.

### Luciferase reporter assay

To investigate the functional role of the putatively inflammation-related differentially methylated region (DMR) located in the *Plakophilin 3* (*PKP3)* gene (chr11:396,135–397,671, 1537bp; hg19), it was cloned into the pCpGfree basic vector (Invivogen) for testing promoter activity and into the pCpGfree promoter vector (Invivogen) for testing enhancer activity **(Supplementary Figure 8)**. To determine the effect of methylation on the activity of the region, *in vitro* methylation of the constructs was performed with *M.Sss*I (NEB). In short, DNA isolated from the blood of a healthy donor was amplified using the Phusion HF polymerase (Thermo Scientific) with primers (Sigma, **Supplementary Table 8)** containing the restriction enzyme sites for *Nsi*I and *Bam*HI at the 5´ends of the forward and reverse primer, respectively. The PCR product clean-up was performed with the ReliaPrep DNA Clean-Up and Concentration kit (Promega). One thousand ng of the amplicon and the plasmid were double digested with 20 U of *Bam*HI (NEB) and 20 U *Nsi*I (NEB) at 37°C for 20 min. The digests were cleaned-up with 0.5x volumes SPRI beads. The ligation was performed with a 1:3 plasmid-to-insert ratio with T4 DNA ligase (NEB) at 16°C overnight. Chemically competent *E. coli* GT115 (pir*^mut^* strain, Invivogen) cells were transformed by performing a heat shock with 5 µl ligation reaction and were spread on LB-zeocin (50 µl/ml; Invivogen) plates. After successful growth in larger volumes, the presence of the insert was verified via colony PCR with Phusion HF polymerase (Thermo Scientific) and short primers targeting the insert region and Sanger sequencing (LGC genomics; **Supplementary Table 8)**. The plasmids containing desired inserts were extracted with the PureYield Plasmid Midiprep System (Promega). *In vitro* methylation of 2 µg constructs was performed with 8 U *M.Sss*I (NEB) at 37°C for 3 h. The enzyme was inactivated at 65°C for 20 min, followed by purification with the ReliaPrep DNA Clean-Up and Concentration kit (Promega).

A549 and H1299 lung epithelial cells were seeded into 24 well plates (1.5×10^5^ cells/well) with Ham F-12 K + 10% foetal bovine serum medium for A549 and RPMI + 10% FBS for H1299 without antibiotics. The next day, 500 ng plasmid and 25 ng pGL4 SV40 control plasmid were co-transfected with Lipofectamine 3000 (Thermo Scientific) and incubated overnight. After cell lysis, the Lucia and Firefly read-out was generated with a Tecan Spark plate reader (Tecan). The Lucia luciferase activity was normalized to the firefly activity. To calculate the fold increase in gene expression, the empty vector was used as a control. Each experiment was performed in triplicates and repeated at least three times. Cells were regularly tested for mycoplasma.

## Results

***Independently of their clinical disease severity and discordance, monozygotic twins with CF are epigenetically highly similar***

The phenotype of CF exhibits extensive inflammation, particularly in the lung [9]. These disease-related processes may have an impact on DNA methylation patterns. In order to investigate CF phenotype- and discordance-related epigenetic alterations in whole blood, 22 clinically well-characterized monozygotic twin pairs with CF from the European CF Twin and Sibling Study were studied [6]. In total, the CF twins comprise eleven different homo- and heterozygous *CFTR* genotypes of which the most abundant is p.Phe508del/p.Phe508del (p.Phe508del homozygous) representing the expected distribution in Europe [84]. From the 44 individuals, peripheral blood DNA was obtained for epigenome-wide DNA methylation analysis of more than 400,000 selected loci in the human genome (Table 1).

The overall methylation patterns in all twins, irrespective of clinical discordance, demonstrate a bimodal distribution, representing the normally observed patterns of DNA methylation values in healthy tissues, with the majority of CpG dinucleotides being either very highly (>85%) or very slightly (<10%) methylated [85]. These results suggest, that even strong inflammatory processes do not shift global DNA methylation patterns in whole blood.

To determine the global effect of clinical severity and discordance on the DNA methylome, the total and pair-wise methylation differences across all loci covered by the array were assessed. Cumulated, all twin pairs revealed a very low mean global intra-pair methylation difference of −0.1% (standard deviation 2.7%) with an upper and lower 95% confidence interval of 0.029% and −0.256%, respectively. Thus, a significant difference cannot be assumed. The pair-wise mean global methylation differences were between −0.6% and 0.4%, the absolute differences were between 1.1% and 2.0% and their extent correlates weakly positively and significantly with clinical intra-pair discordance (Delta) (r^2^ = 0.378, p-value = 0.023) and age (r^2^ = 0.391, p-value = 0.009). This confirms other studies demonstrating, that monozygotic twins are epigenetically highly similar and reveals that this high similarity remains even in individuals clinically discordant for a severe inflammatory disease such as CF [21,86,87].

### Differences in blood cell composition in CF twins are minor and occur mainly in discordant pairs

**Figure 1.**
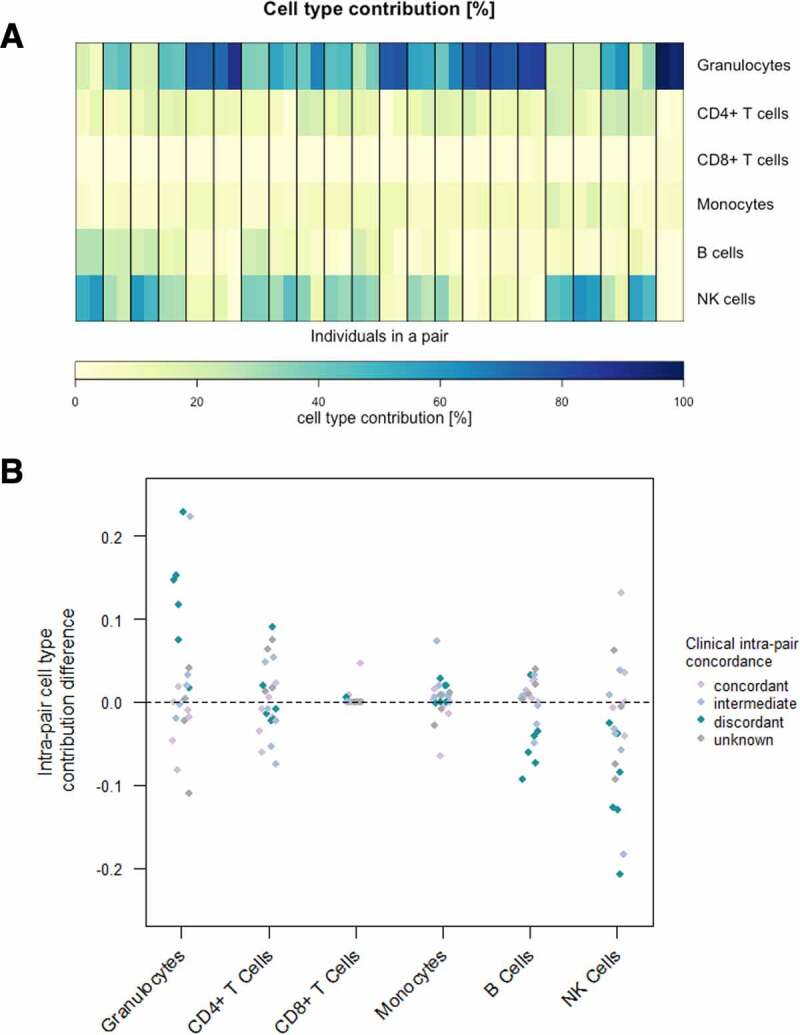
**Blood cell-type contribution in CF twins based on whole blood DNA methylation**. (a) Estimated cell-type distribution (%) of six different cell-types in peripheral blood according to DNA methylation values in the 44 subjects representing 22 twin pairs. Heatmap of the contributions of six different cell-types in peripheral blood. Twin pairs are separated by black vertical lines. The twin with the milder phenotype is displayed on the left, whereas the one with the more severe phenotype is shown on the right. (b) Scatterplot of intra-pair cell-type contribution differences for six different cell-types. Clinical intra-pair discordance is indicated in colour.

Blood cell-type composition is known to change upon inflammation [88]. Hence, the blood cell contribution of six different blood cells (granulocytes, CD4^+^ T cells, CD8^+^ T cells, B cells, monocytes and natural killer cells) has been estimated for all samples based on the obtained DNA methylation values using six reference methylomes and an algorithm developed by Houseman et al. [75,82]. Irrespective of the individual phenotypical severity and the intra-pair clinical discordance, blood cell compositions within one twin pair are noticeably similar. The minor intra-pair cell-type contribution differences occur mainly in discordant twin pairs and apparently display a shift from natural killer cells to granulocytes in the more severely affected individuals (Figure 1**a**, SupplementaryFigure 1**a, b)**. This is in concordance with the fact, that a high abundance of neutrophils is frequently observed in individuals with cystic fibrosis and points out that despite clinical discordance, the blood cell-type composition differences in monozygotic CF twins are surprisingly small [88].

***Monozygotic CF twin pairs exhibit only a few differentially methylated positions, correlating with disease severity and intra-pair discordance***

**Figure 2.**
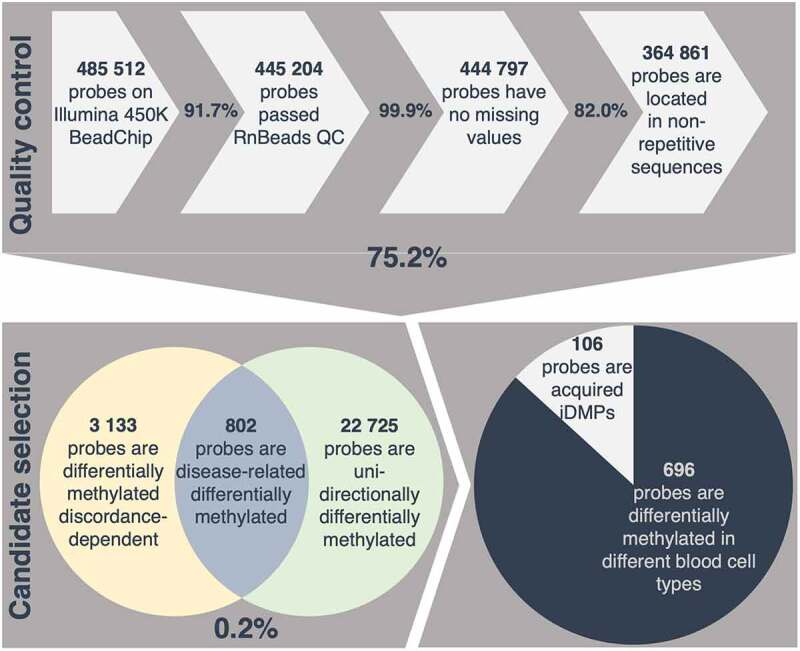
**Selection process applied for identification of acquired inflammation-related differentially methylated positions (iDMPs)**. 75.2% of all probes measured passed a strict quality control (QC) including removal of cross-reactive and unreliable probes, probes with missing values and probes located in repetitive sequences. 0.2% of all retained probes are considered disease-related differentially methylated according to the assumptions described in the methods. Given that 87% of iDMPs are significantly differentially methylated in different blood cell-types, 106 probes are presumed to be acquired iDMPs.

In order to investigate disease-related methylation alterations, a selection process was developed and applied to identify CpG sites which are differentially methylated in an intra-pair comparison, taking into account individual severity and intra-pair discordance. 75.2% of all investigated positions covered passed a strict quality control with removal of unreliable probes, probes with missing values and probes located in repetitive sequences. Only 0.86% of all positions investigated, present discordance-dependent differential methylation according to the defined requirements. Of these, 25.6% are consistently differentially methylated in one direction in a pair-wise comparison (e.g., either consistently hyper- or hypo-methylated in all of the more severely affected individuals of a twin pair) resulting in 802 disease-related differentially methylated positions (DMPs). Due to the strongly inflammatory component of CF, these positions are referred to as putative inflammation-related DMPs (iDMPs) (Figure 2**, Supplementary table 5)**.

***A high proportion of disease-related differentially methylated positions may reflect blood cell-type composition effects***

Since DNA methylation is known to be tissue- and cell-type specific, the list of candidate iDMPs was examined for known blood cell-type specific differentially methylated positions from a publicly available dataset [75,89]. Interestingly, 86.8% of iDMPs have been shown to be significantly differentially methylated between different blood cell-types and therefore could reflect differential blood cell counts between the twin pairs. This would be in concordance with CF being a disease characterized by massive chronic inflammation, especially in the lung with consequent alterations in the immune cell composition and underscores the applied selection process for inflammation-related differentially methylated positions [90].

The remaining 106 iDMPs are considered acquired in relation with the CF disease severity, which may be environmentally promoted and accordingly of particular interest for diseases [91] (Figure 2**, Supplementary table 6)**.

### Disease-related methylation differences in monozygotic twins are of a small magnitude

**Figure 3.**
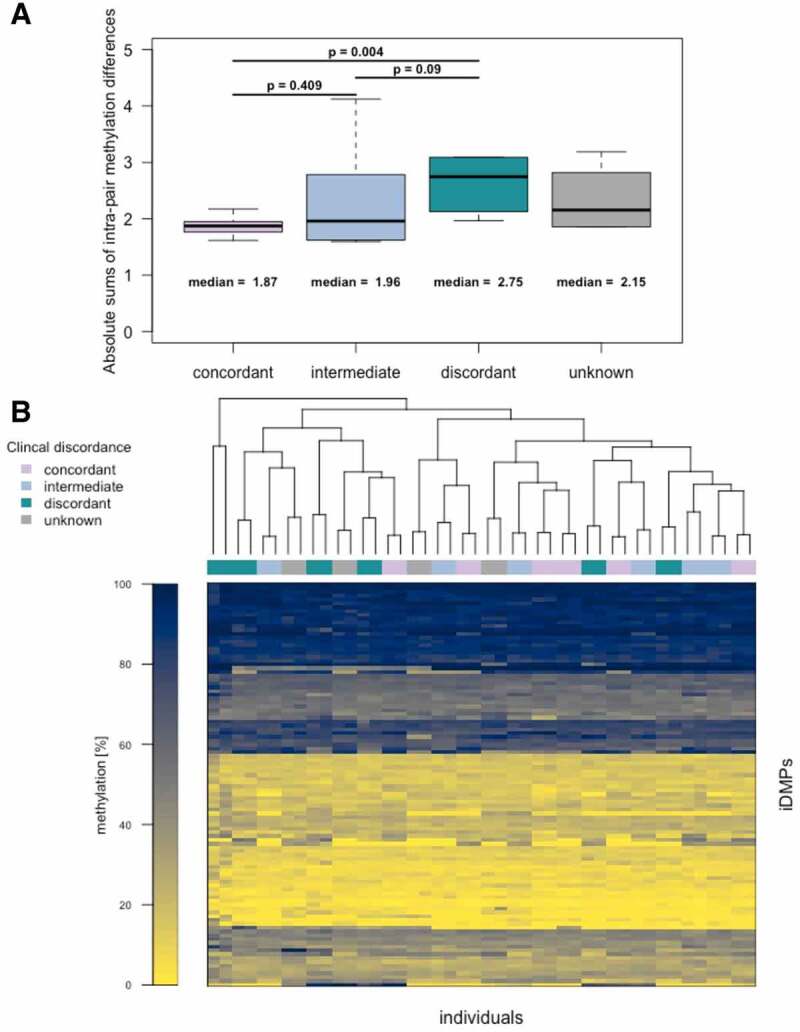
**Methylation and intra-pair methylation differences in CF twins**. (a) Absolute intra-pair methylation differences of putative acquired iDMPs. Clinical discordance is indicated in colour. Methylation differences are significantly higher (p = 0.004, one-sided Wilcoxon test) in discordant than in concordant twin pairs. The means and the medians of the absolute sums of the differences increase with the extent of clinical discordance. (b) Heatmap of methylation [%] in candidate iDMPs in 44 individuals. Rows and columns are clustered by complete hierarchical clustering and Euclidean distance.

To validate the applied selection process, and thus the relation of the 106 selected candidate positions with the clinical discordance of the twin pairs, the extent of differential methylation was assessed. A trend from lower methylation differences to higher ones with increasing discordance can be observed for the selected iDMPs. Interestingly, twin pairs with unknown discordance exhibit the same mean of absolute sums of intra-pair methylation differences as twin pairs with intermediate discordance. Furthermore, concordant twin pairs display significantly (p = 0.004, one-sided Wilcoxon test) lower sums of intra-pair methylation differences than discordant twin pairs (Figure 3a). This confirms that the putative iDMPs are differentially methylated in a discordance-dependent manner as expected due to this type of selection process. However, when investigating the DNA methylation values in the twins, there are small differences between the individuals and the pairs clearly cluster together (Euclidean distance and complete hierarchical clustering), emphasizing the epigenetic similarity of twins even in the event of clinical discordance (Figure 3b).

***Acquired inflammation-related differentially methylated positions are not enriched in GO-terms and do not cluster at genomic regions***

In order to determine whether alterations in DNA methylation which are related to the disease phenotype of CF may accumulate in certain genomic or functional regions, enrichment analysis of the candidate iDMPs was performed. Acquired iDMPs occur to be relatively evenly distributed across the chromosomes. In comparison to all CG dinucleotides measured, they are not enriched in imprinted or (sub)telomeric regions. They do not accumulate in CG-rich or regulatory regions such as enhancers, DNA hypersensitivity sites or promoters. Also, no accumulation at gene bodies, transcription start sites or untranslated regions was observed. Of 106 iDMPs, 35 are located in a gene promoter and 47 in a gene body (including pseudogenes and antisense transcripts) and 21, 19 and 10 are located in a CpG island, CpG shore and CpG self, respectively (SupplementaryFigure 2). These 82 gene-associated iDMPs correspond to 81 inflammation-related differentially methylated regions (iDMRs) and 79 different genes.

Gene ontology (GO) enrichment analysis was performed with gene-associated iDMPs, however it did not deliver significant (Fisher´s Exact test, FDR) enrichment for the biological processes, the molecular function or the cellular component of the respective genes. In summary, no positional or functional hotspots or patterns were observed for acquired iDMPs; however, due to the stringent selection process, only few observations could be included into the analyses, which makes it difficult to notice genome-wide patterns.

***An acquired iDMR in a CpG island in the PKP3 gene body is highly variably methylated in blood from lung cancer patients compared to healthy controls***

It has been shown that environmental factors, such as cigarette smoking and inflammation can change DNA methylation patterns. These alterations can have an impact on the risk, the development and the prognosis of diseases such as cancer, which is known to be strongly associated with environmental factors (i.e., smoking) [22, 23, 92, 93, L. 24].

Since chronic inflammation is a major risk factor for cancer in general, putative inflammation-related variations in DNA methylation may be relevant for lung cancer. To identify positions which are of particular interest in the context of inflammation and lung cancer, the list of putative acquired iDMPs was inspected a) for the loci most prominently differentially methylated in the CF twin cohort and b) for loci for which methylation differences were also observed in TCGA in lung cancer tissues compared to normal adjacent tissues. Of 106 iDMPs, 11 were strongly (more than once ≥10%) or frequently (more than four times ≥5%) differentially methylated in the CF twin data and 21 were differentially methylated (≥5%) in LUAD and LUSC compared to the corresponding normal tissue in TCGA data, with an overlap of three iDMPs in these two groups. Two of the candidates are located in the same region (13) directly adjacent to each other in the gene body of *Plakophilin 3* (*PKP3)* (SupplementaryFigure 3).

DNA methylation of these selected acquired iDMPs and flanking regions was assessed in 218 whole blood samples from a lung cancer case-control study (Table 2). Of these 29 most interesting acquired iDMPs with regard to lung cancer, 21, corresponding to 20 genomic regions could be tested with bisulphite targeted-NGS in whole blood from lung cancer patients and healthy controls. The power to detect differences in mean methylation levels of at least 5% in the lung cancer case-control cohort of 109 pairs for all positions tested is above 99% **(Supplementary table 4)**.

To cover all samples, two flow cells (MiSeq, Illumina) were loaded. A mean cluster density of 776 K/mm^2^ could be achieved of which 94.66% (36.19 million reads of 38.27 million reads) passed the filter. On average, 80.62% of the bases hold a quality score above Q30. In order to obtain only high-quality sequences, on average 98.01% and 88.29% of all reads were trimmed from the forward and reverse reads, respectively. No reads were removed. Forward reads had a better quality than reverse reads which is generally observed in paired-end sequencing with Illumina (SupplementaryFigure 4**A**). Of all reads, 62% could be assigned to the samples. We assume that approximately 25% of the library is PhiX spike-in. Of all reads obtained for the 218 samples, 94.65% passed the filter and could be processed with the methylation analysis pipeline ‘bicycle.’ 84.55% of all reads which passed the filter could be uniquely aligned to the human genome. 0.86% were mapped to both Watson (+) and Crick (-) strands, 2.74% were aligned to multiple regions in the genome and 5.57% of the reads were removed due to possibly failed bisulphite-conversion (SupplementaryFigure 4b). 98.55% of the uniquely aligned high-quality reads could be aligned on target (i.e., at the targeted regions) (SupplementaryFigure 4c). For all samples, a mean coverage of 1694x could be achieved across all regions of interest. No region had a mean coverage below 100x with an average of 1634x (range: 124x – 4841x) (SupplementaryFigure 4d).

**Figure 4.**
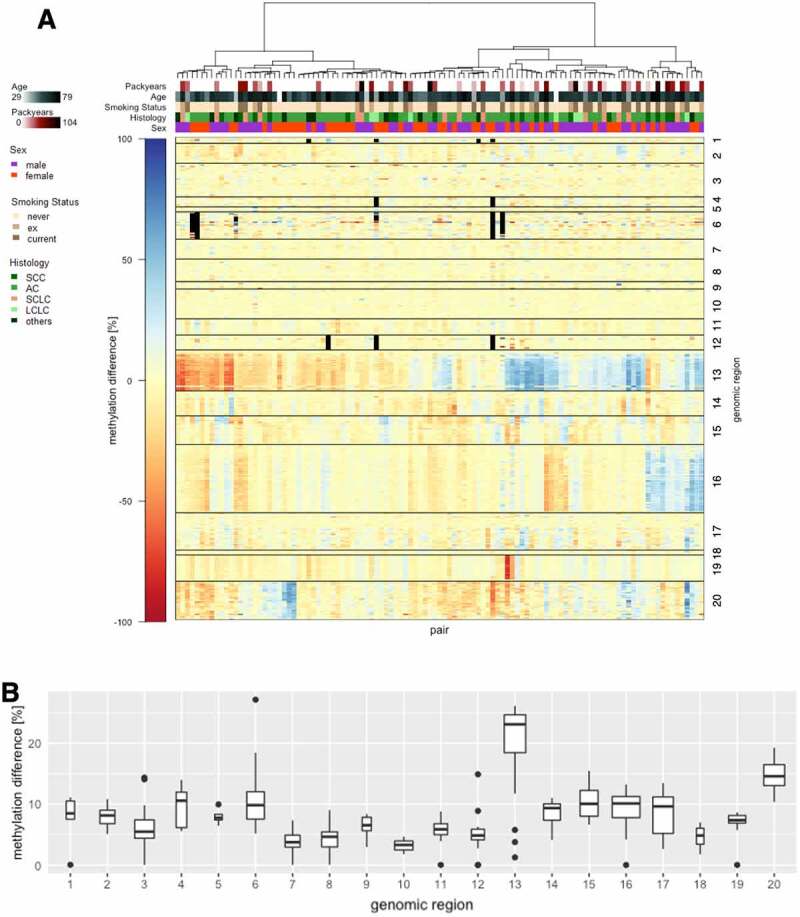
**DNA methylation differences in lung cancer cases compared to healthy controls**. (a) Heatmap of methylation differences in CG dinucleotides of 20 genomic regions (numbers), corresponding to 21 iDMPs (rows separated by black lines) in 209 pairs (columns). NAs are shown in black, clinical parameters are colour coded (b) Bar plot of the means of all absolute intra-pair methylation differences [%] per CG dinucleotide of the 20 numbered regions. Median values across the regions are indicated. The width of the boxes represents the number of observations (i.e., the number of CG dinucleotides per region).

Methylation differences between lung cancer case-control pairs are small in most regions. If differential methylation occurs, then it is region-specific with respect to extent and direction, implying that in one pair, the whole region is consistently differentially methylated (Figure 4a). Applying a two-sided, paired Student´s t-test, no significant differences of the tested iDMPs in lung cancer compared to controls could be observed after FDR correction of p-values. Principal component analysis did not reveal any clustering according to known clinical parameters (Supplementary Figure 5). Further, correlation and multiple regression analysis of mean methylation values per region and known clinical parameters of the samples did not disclose any relations (correlation and coefficient of determination values below 0.18) between methylation in the investigated regions and known clinical parameters (sex, cancer occurrence, cancer histology, age, packyears and smoking status). However, of 20 regions analysed, one region (#13) comprising two adjacent candidate iDMPs (cg17840408, cg25258098) appears to be highly variably methylated across the lung cancer-case control cohort in both directions (i.e., hyper- and hypo-methylated) irrespective of known clinical parameters (Figure 4b**, Supplementary Figure 6)**. The iDMR of interest is located on a CpG island in the gene body of *PKP3*, encoding for the desmosomal protein PKP3, known to play a role in carcinogenesis [94]. It overlaps with a putative regulatory region **(Supplementary Figure 7)**. CpG islands are often regulated via DNA methylation [83,95–98]. In contrast to promoter methylation, gene body methylation is more frequently, associated with active gene expression and may prevent cryptic transcription initiation [99,100]. Upregulation of gene expression by intragenic CpG island hypermethylation has been demonstrated to be associated with cancer [101]. Also, variably methylated regions have been shown to be enriched for enhancer histone modifications and transcription factor-binding sites and may exhibit important regulatory functions especially regarding cell- and tissue-type [102]. It is therefore suggested that this iDMR, hypermethylated in CF and highly variably methylated in lung cancer and healthy subjects, may have functional relevance in the context of lung inflammation and cancer.

**Figure 5.**
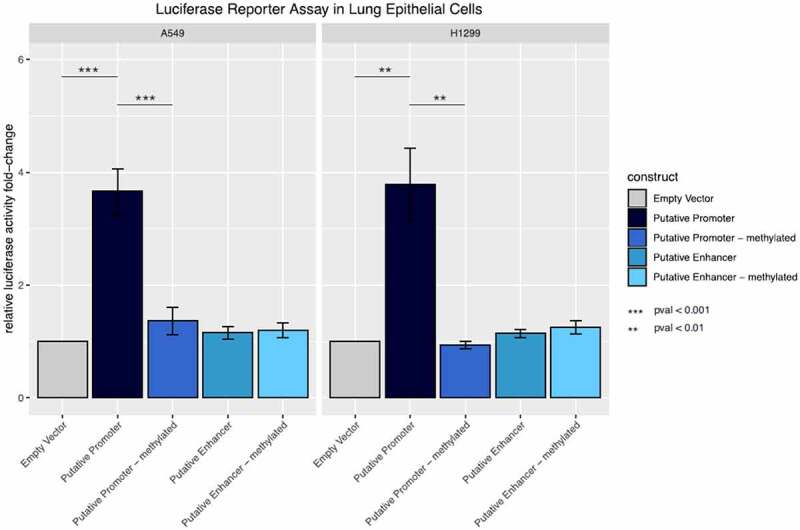
**Promoter and enhancer activity of the methylated and unmethylated inflammation-related DMR in lung epithelial cells**. Fold change relative luciferase activity in A549 and H1299 cells. The empty vector corresponds to a construct without promoter sequence. For the putative promoter vector, the sequence of interest has been cloned into the empty vector upstream of the luciferase gene. For the putative enhancer construct, the region of interest was cloned upstream of a minimal promoter for the luciferase gene. Both vectors were compared to their *in silico* methylated counterparts.

***The candidate iDMR in a CpG island in the*** **PKP3 *gene body exhibits a methylation-dependent promoter activity in lung epithelial cells***

Evaluation of publicly available chromatin immunoprecipitation DNA-sequencing (ChIP-seq) data from the Broad institute and ENCODE revealed that 5´-adjacent to the candidate region in the *PKP3* gene body is a CTCF binding site and that the iDMR may be located in an insulator in NHLF, HUVEC and H1-hESC cells; however, it could function also as a promoter for instance in HepG2 cells [77–80]. CTCF binding facilitates inter- and intra-chromosomal chromatin looping and thus enables physical proximity of regulatory regions. Further, CTCF binding sites colocalizes with promoters and enhancers and attracts or repels transcription factors and subsequently activates or represses transcription. CTCF preferentially binds to unmethylated CTCF binding sites and, in turn, binding of CTCF to insulator regions prevents its methylation [103–105].

DNA methylation of *cis* and *trans* regulatory functional elements, such as CTCF binding sites, impacts their function and subsequently results in activation or inactivation of gene expression in a context- and cell-type specific manner [32,106]. To evaluate the functional relevance of the putative regulatory region, associated with a CTCF binding site in the tissue of interest, luciferase reporter assays were performed in the lung epithelial cells A549 and H1299. Therefore, the region of interest was cloned into a CpG-free vector upstream of the luciferase gene with and without minimal promoter to determine functional activity as enhancer and promoter, respectively **(Supplementary Figure 8)**. In both cell lines a highly significant increase in the relative luciferase activity (Welch Two Sample t-test, p-value = 0.0008513) could be shown for constructs containing the region of interest 5´ to the luciferase gene compared to vectors without the respective region. This indicates that the putative regulatory region functions as a promoter in lung epithelial cells. However, the insertion of the same regulatory element together with a minimal promoter did not result in enhanced luciferase activity in comparison to the empty plasmid, suggesting that it does not function as an enhancer in the tested cell lines. To determine the effect of DNA methylation of the regulatory region on luciferase expression, both constructs were *in vitro* methylated. Since the vectors are CpG free, only the inserted sequence is methylated. While no change in luciferase activity was shown for the methylated (A549: 1.20 ± 0.129; H1299 :1.25 ± 0.117) and unmethylated (A549: 1.15 ± 0.109; H1299 :1.14 ± 0.072) enhancer constructs, methylation of the promoter construct resulted in significantly decreased luciferase activity (A549: −3.66 ± 0.400; H1299: −3.78 ± 0.0644) compared to the unmethylated region (A549: −1.36 ± 0.0239; H1299: −0.94 ± 0.064) suggesting that the promoter function of the region of interest is depleted upon its methylation (Figure 5).

Taken together, these results show that the iDMR located in the *PKP3* gene body can function as a promoter in lung epithelial cells and that this function is eliminated via DNA methylation.

## Discussion

In order to investigate inflammation-related aberrant DNA methylation, genome-wide methylation was analysed in twins with CF, as a chronic inflammatory lung disease. CF disease severity is highly associated with the degree and the duration of inflammatory processes, particularly in the lung [4].

In the lung, the neutrophilic inflammation leads to release of neutrophil elastases, matrix-metalloproteinases, nitric oxide and reactive oxygen species (ROS), which cannot be efficiently resolved and cause massive tissue injury. Therefore, the CF lung is flooded with proinflammatory mediators such as tumour necrosis factor alpha (TNF-α), interleukin (IL)-1β, IL-6, IL-8 and others. On the other hand, anti-inflammatory cytokines for instance, IL-10, are decreased, resulting in impaired resolution of inflammation and an extensive and chronic inflammatory state including defective apoptosis and phagocytosis [5,107]. Like CF, COPD is characterized by mucus hypersecretion, oxidative stress, neutrophilic inflammation and the involvement of macrophages. TNF‐α, IL‐1β, IL‐6 and IL‐8 are contributing to inflammation in COPD as well [108]. COPD is an inflammatory disease of the lung highly associated with lung cancer [55,109]. COPD and other chronic inflammatory disorders of the lung are associated with an increased risk for lung cancer development [53, J. 54, 55]. Also, the major risk factor for lung cancer, smoking, is known to trigger lung inflammation [110–112].

CF, although a disease characterized by massive chronic lung inflammation, is not associated with lung cancer risk [58]. This may be due to the fact that the early and strong impairment of the lung is fatal, or lung transplantation is indicated and performed prior to the development of lung cancer [113,114]. It has been even suggested that the p.Phe508del deletion has a protective role in lung cancer [115]. However, due to its severe phenotype, we consider CF an appropriate model for investigating excessive and persistent chronic inflammation of the lung with potential implications for lung cancer. Since the *CFTR* genotype, the genomic background, sex, age and socioeconomic factors can be neglected in this study exploring monozygotic twins, we assume that clinical intra-pair discordance is at least to some extent due to a different degree of inflammation [84,116,117].

Global alterations of DNA methylation patterns have been described in cancer and ageing [118,119]. Investigating the global methylome of monozygotic twins, no overall alterations in DNA methylation were observed in intra-pair comparisons. These observations were independent of clinical discordance of the respective twin pair and are in accordance with previous studies, showing that monozygotic twins are epigenetically highly similar and emphasize the importance of age, sex, genetic background and environment for the shape of the methylome [86,87,120–126].

Global and locus-specific DNA methylation differences as well as phenotypic discordance in monozygotic twins are known to increase with age [127,128]. In this study, no relation between the absolute global methylation difference and the age of the twin pairs could be observed. However, the twins evaluated in this study are relatively young with a mean age of 14.4 years.

DNA methylation is known to be cell-type-specific. Therefore, DNA methylation values obtained from an epigenome-wide analysis can be applied to estimate the cell-type proportion in a sample with a mixed cell population [75,82]. Our data indicate high similarities in blood cell proportion in intra-pair comparisons. Cell-type composition differences were mainly observed in clinically discordant twins which is in accordance with the fact that inflammatory diseases alter the abundance of immunological cells in blood [129]. Relative differential cell-type counts are mainly attributed to granulocytes and natural killer cells in an inverse manner. A larger fraction of granulocytes in blood from more severely diseased individuals, compared to their less severely affected twin counterparts, may point to neutrophilia, the high abundance of neutrophils (granulocytes, basophils and eosinophils), in the blood and subsequently the lung which in turn is a well-known symptom in CF [88].

To discover whether chronic lung inflammation may cause locus-specific differential methylation in the blood, positions differentially methylated in a severity- and discordance-specific manner were selected. Thus, a subset of putative iDMPs could be identified. Our data indicate that a large proportion of these iDMPs have been shown to be differentially methylated in a blood cell-type-specific manner and hence may be attributed to cell-type composition effects [75,89]. Given that the abundance of immune cells in the blood is likely to be altered in an inflammatory setting, this emphasizes that the selected loci may be related to inflammation [129].

All other iDMPs are considered to be acquired due to exposure to the inflammatory environment. Acquired alteration of DNA methylation induced by environmental factors is of particular interest in the context of diseases [91,130,131]. The 106 iDMPs we observed in our study are not enriched in genomic location and in relation to particular genes or to functional regions. Seventy-seven percent of the iDMPs are considered gene-associated; however, their respective genes are not enriched in biological process, molecular function or cellular components. However, due to the stringent selection process, the number of observed iDMPs may have been too small for these analyses.

Among the 21 tested loci, none was differentially methylated in blood from lung cancer cases compared to healthy controls. However, one DMR including two iDMPs, cg17840408 and cg25258098, directly adjacent to each other and hyper-methylated in blood of the CF twins, was found to be highly variably methylated in whole blood of lung cancer cases compared to healthy controls, irrespective of known clinical parameters (sex, age, packyears, current smoking status and lung cancer histology in cases) which are known to be related to DNA methylation [24,121,132,133]. This variable methylation is extended also to the 5´- and 3´-flanking regions. The iDMR is located on chromosome 11p15.5, a subtelomeric region, in a CpG island indicating a regulatory function of DNA methylation in this region since these CpG dense regions are often prone to regulation by DNA methylation [83,95–98].

In non-related individuals, one major cause of interindividual methylation variability are genetic differences, whereas in monozygotic twins it is determined either by stochastic effects, which result in an epigenetic drift or by environmental exposure, such as chronic inflammation [122,128]. It has been shown that DNA methylation at certain CpG sites differs between distinct tissues, whereas others are consistently methylated in any tissue of an individual but display interindividual differences. The latter may be particularly valuable in the context of risk biomarkers in peripheral blood independently of functional relevance as they may offer information regarding epigenetic regulation in less accessible tissues [134].

ChIP-seq data from the Broad institute and ENCODE revealed that in lung cells the putatively inflammation-related differentially methylated region may function as an insulator, whereas in other cell-types it may have promoter activity [77–80]. CTCF binding sites regulate inter- and intra-chromosomal interactions between functional elements, enable three-dimensional chromatin structure, separate topological regions and regulate transcription factor accessibility. Therefore, CTCF binding can dynamically control gene transcription and alternative splicing events and thus contributes to dynamic processes such as cell differentiation and generation of immune cell diversity [103,105]. Our results indicate, that the iDMR functions as a promoter in lung epithelial cells, which is inactivated upon methylation.

The putatively regulatory iDMR, overlapping with a CTCF-binding site is located in the gene body of *PKP3* (NM_007183). *PKP3* encodes for the 81.081kD PKP3 protein which is expressed in the desmosomes and the nucleus of the simple and the most stratified epithelia and plays a role in linking cadherins and intermediate filaments in the cytoskeleton, in cell–cell communication by enhancing the desmosomal strength, in the RNA metabolism and as transcription factor associated with cancer and metastasis [135–139]. Desmosomal proteins such as PKP3 have implications in epithelial integrity and tissue remodelling, both hallmarks of inflammatory lung diseases, such as CF [140,141]. They further appear to have tumour-promoting as well as tumour-suppressing properties in distinct cancer entities. They are essential for epithelial homoeostasis, tissue integrity and regulation of cell proliferation, differentiation, migration, invasion and apoptosis and are regulated by growth factors [94,142].

Altered expression of *PKP3* transcripts has already been described in connection with lung cancer and other cancer types. Depending on the type of cancer, over-expression of PKP3 protein and *PKP3* mRNA have been shown to have oncogenic and tumour suppressing effects [94,143–146]. In non-small cell lung tumours, which include lung adenocarcinomas and squamous cell carcinomas, *PKP3* is described as an oncogene, which has the potential as a prognostic marker but also in the therapeutic area [147]. Desmosomal loss and loss of haematopoietic PKP3 is associated with increased inflammation [136,148,149]. Overexpression of adhesion molecules such as PKP3 could be demonstrated to be associated with differential infiltration of various immune cells in human lung cancer tissue, making the demosome status a potential biomarker in the context of immunotherapies [150].

Also, in the airway epithelia of CF lungs, epithelial junctions and integrity may have major roles in permeability to ions and pathogen products and subsequently lung inflammation [151].

Interestingly, aberrant methylation and expression of *PKP3* has been associated with endometriosis, which is an inflammatory disease of the endometrium and has been observed in the mucosa of ulcerative colitis patients with and without cancer [152–154].

Directly adjacent to *PKP3* is the *single immunoglobin interleukin-1 (IL-1)-Related Receptor* (*SIGIRR*) gene, which is also known as *IL-1 receptor 8* (*IL-1R8*). *IL-1R8* is anti-inflammatory and shows its effect against inflammatory diseases, also in the lung [L. 155].

It is interesting to note that frequent polymorphisms in the region comprising *PKP*3, *SIGIRR* and another gene, *TMEM16J*, have been linked to susceptibility to tuberculosis, another inflammatory lung disease [156].

At present it is not yet possible to say for certain which genes in which cell-types are regulated by the methylation-dependent promoter iDMR. However, due to the functional studies performed, it can be hypothesized that this iDMR acts as an alternative promoter for the *PKP3* gene.

In summary, a unique cohort of monozygotic CF twins has been clinically and epigenetically characterized in this study. The remarkable epigenetic intra-pair similarity observed even in clinically discordant twins, indicates that besides genetic and epigenetic phenotype-contribution further factors, such as the microbiome, that is likewise related to CF disease severity and progression, have a major impact on the individual development of cystic fibrosis [157,158]. Selected positions could be shown to be differentially methylated in CF twins in a discordance-dependent manner. These could reflect a consequence of chronic lung inflammation for the DNA methylome in the blood or may have a functional effect on the phenotype. Additionally, we observed one inflammation-related differentially methylated region to be highly variably methylated in the whole blood of individuals with and without lung cancer. Functional assays revealed that this region functions as a methylation-dependent promoter in lung epithelial cells. This suggests that the region in the *PKP3* gene body is a functional promoter in lung tissues which is hypermethylated upon inflammation and variably methylated in the context of lung cancer. Whether this regulatory region may act as an alternative promoter for *PKP3* gene or has insulatory or chromatin remodelling function, thus influencing nearby genes such as *SIGIRR* and *TMEM16J in situ*, may be elucidated in further research.

A better understanding of the effect of chronic inflammation on DNA methylation and the presentation of inflammation-related epigenetic changes in lung cancer may improve existing strategies in risk assessment of inflammatory lung diseases and lung cancer. Thus, it can provide further insights into the mechanisms of carcinogenesis promoted by inflammation, such as epithelial to mesenchymal transition and epithelial tissue integrity. These insights have the potential to pave the way for advances in risk stratification, prognosis and treatment of inflammation-related diseases and cancer.

## Supplementary Material

Supplemental Material

## Lung cancer case-control cohort

All participants gave their informed consent. The study was approved by the ethical committee of the Medical Faculty of the University of Heidelberg (Nr. 182/96, 201/98 and 199/2001).

## References

[1] Riordan JR, Rommens JM, Kerem B, et al. Identification of the cystic fibrosis gene: cloning and characterization of complementary DNA. Science. 1989;245(4922):1066–1073. 2475911 10.1126/science.2475911

[2] Warwick WJ. The incidence of cystic fibrosis in Caucasian populations. Helv Paediatr Acta. 1978;33(2):117–125. 659256

[3] Stanke F, Tümmler B. Classification of CFTR mutation classes. Lancet Respir Med. 2016;4(8):e36. 27377412 10.1016/S2213-2600(16)30147-3

[4] Castellani C, Assael BM. Cystic fibrosis: a clinical view. Cell Mol Life Sci. 2017;74(1):129–140. 27709245 10.1007/s00018-016-2393-9 PMC11107741

[5] Roesch EA, Nichols DP, Chmiel JF. Inflammation in cystic fibrosis: an update. Pediatr Pulmonol. 2018;53(S3):S30–S50. 29999593 10.1002/ppul.24129

[6] Mekus F, Ballmann M, Bronsveld I, et al. Categories of ΔF508 homozygous cystic fibrosis twin and sibling pairs with distinct phenotypic characteristics. Twin Res. 2001;3(4):277–293. 10.1375/136905200320565256 11463149

[7] Brownell JN, Bashaw H, Stallings VA. Growth and Nutrition in Cystic Fibrosis. Semin Respir Crit Care Med. 2019;40(6):775–791. 31659726 10.1055/s-0039-1696726

[8] Cantin AM, Hartl D, Konstan MW, et al. Inflammation in cystic fibrosis lung disease: pathogenesis and therapy. J Cyst Fibros. 2015;14(4):419–430. 25814049 10.1016/j.jcf.2015.03.003

[9] Cutting GR. Cystic fibrosis genetics: from molecular understanding to clinical application. Nat Rev Genet. 2015;16(1):45–56. 25404111 10.1038/nrg3849 PMC4364438

[10] Gong J, Wang F, Xiao B, et al. Genetic association and transcriptome integration identify contributing genes and tissues at cystic fibrosis modifier loci. PLoS Genet. 2019;15(2):e1008007. 30807572 10.1371/journal.pgen.1008007 PMC6407791

[11] Shanthikumar S, Neeland MN, Saffery R, et al. Gene modifiers of cystic fibrosis lung disease: a systematic review. Pediatr Pulmonol. 2019;54(9):1356–1366. 31140758 10.1002/ppul.24366

[12] Stanke F, Hector A, Hedtfeld S, et al. An informative intragenic microsatellite marker suggests the IL-1 receptor as a genetic modifier in cystic fibrosis. Eur Respir J. 2017;50(6):6. 10.1183/13993003.00426-2017 29284683

[13] Bolund ACS, Starnawska A, Miller MR, et al. Lung function discordance in monozygotic twins and associated differences in blood DNA methylation. Clin Epigenetics. 2017;9(1):132. 29299071 10.1186/s13148-017-0427-2 PMC5740718

[14] Collaco JM, Blackman SM, McGready J, et al. Quantification of the relative contribution of environmental and genetic factors to variation in cystic fibrosis lung function. J Pediatr. 2010;157(5):802–807.e801-803. 10.1016/j.jpeds.2010.05.018 PMC2948620 20580019

[15] Tan Q, Christiansen L, von Bornemann Hjelmborg J, et al. Twin methodology in epigenetic studies. J Exp Biol. 2015;218(Pt 1):134–139. 25568460 10.1242/jeb.107151

[16] Xiang Z, Yang Y, Chang C, et al. The epigenetic mechanism for discordance of autoimmunity in monozygotic twins. J Autoimmun. 2017;83:43–50. 28412046 10.1016/j.jaut.2017.04.003

[17] Generali E, Ceribelli A, Stazi MA, et al. Lessons learned from twins in autoimmune and chronic inflammatory diseases. J Autoimmun. 2017;83:51–61. 28431796 10.1016/j.jaut.2017.04.005

[18] Gomez-Cabrero D, Almgren M, Sjöholm LK, et al. High-specificity bioinformatics framework for epigenomic profiling of discordant twins reveals specific and shared markers for ACPA and ACPA-positive rheumatoid arthritis. Genome Med. 2016;8(1):124. 27876072 10.1186/s13073-016-0374-0 PMC5120506

[19] Hwang JY, Lee HJ, Go MJ, et al. Genome-wide methylation analysis identifies ELOVL5 as an epigenetic biomarker for the risk of type 2 diabetes mellitus. Sci Rep. 2018;8(1):14862. 30291282 10.1038/s41598-018-33238-9 PMC6173741

[20] Roos L, van Dongen J, Bell CG, et al. Integrative DNA methylome analysis of pan-cancer biomarkers in cancer discordant monozygotic twin-pairs. Clin Epigenetics. 2016;8(1):7. 26798410 10.1186/s13148-016-0172-y PMC4721070

[21] Schamschula E, Hagmann W, Assenov Y, et al. Immunotyping of clinically divergent p.Phe508del homozygous monozygous cystic fibrosis twins. J Cyst Fibros. 2021;20(1):149–153. 32540173 10.1016/j.jcf.2020.06.009

[22] Fasanelli F, Baglietto L, Ponzi E, et al. Hypomethylation of smoking-related genes is associated with future lung cancer in four prospective cohorts. Nat Commun. 2015;6(1):10192. 26667048 10.1038/ncomms10192 PMC4682166

[23] Wrage M, Hagmann W, Kemming D, et al. Identification of HERC5 and its potential role in NSCLC progression. Int J Cancer. 2015;136(10):2264–2272. 25353388 10.1002/ijc.29298

[24] Baglietto L, Ponzi E, Haycock P, et al. DNA methylation changes measured in pre-diagnostic peripheral blood samples are associated with smoking and lung cancer risk. Int J Cancer. 2017;140(1):50–61. 27632354 10.1002/ijc.30431 PMC5731426

[25] Bergougnoux A, Claustres M, De Sario A. Nasal epithelial cells: a tool to study DNA methylation in airway diseases. Epigenomics. 2015;7(1):119–126. 25687471 10.2217/epi.14.65

[26] Farré P, Jones MJ, Meaney MJ, et al. Concordant and discordant DNA methylation signatures of aging in human blood and brain. Epigenetics Chromatin. 2015;8(1):19. 25977707 10.1186/s13072-015-0011-y PMC4430927

[27] Houseman EA, Kim S, Kelsey KT, et al. DNA Methylation in Whole Blood: uses and Challenges. Curr Environ Health Rep. 2015;2(2):145–154. 26231364 10.1007/s40572-015-0050-3

[28] Huang Y-T, Chu S, Loucks EB, et al. Epigenome-wide profiling of DNA methylation in paired samples of adipose tissue and blood. Epigenetics. 2016;11(3):227–236. 26891033 10.1080/15592294.2016.1146853 PMC4854552

[29] Um SW, Kim Y, Lee BB, et al. Genome-wide analysis of DNA methylation in bronchial washings. Clin Epigenetics. 2018;10(1):65. 29796116 10.1186/s13148-018-0498-8 PMC5960087

[30] Choi J, Lyons DB, Kim MY, et al. DNA Methylation and Histone H1 Jointly Repress Transposable Elements and Aberrant Intragenic Transcripts. Mol Cell. 2020;77(2):310–323.e317. 31732458 10.1016/j.molcel.2019.10.011

[31] Pfeifer GP. Defining Driver DNA Methylation Changes in Human Cancer. Int J Mol Sci. 2018;19(4):1166. 10.3390/ijms19041166 PMC5979276 29649096

[32] Tirado-Magallanes R, Rebbani K, Lim R, et al. Whole genome DNA methylation: beyond genes silencing. Oncotarget. 2017;8(3):5629–5637. 27895318 10.18632/oncotarget.13562 PMC5354935

[33] Wolff F, Leisch M, Greil R, et al. The double-edged sword of (re)expression of genes by hypomethylating agents: from viral mimicry to exploitation as priming agents for targeted immune checkpoint modulation. Cell Commun Signal. 2017;15(1):13. 28359286 10.1186/s12964-017-0168-z PMC5374693

[34] Calle-Fabregat CDL, Morante-Palacios O, Ballestar E. Understanding the Relevance of DNA Methylation Changes in Immune Differentiation and Disease. Genes (Basel). 2020;11(1):110. 10.3390/genes11010110 PMC7017047 31963661

[35] Ciechomska M, Roszkowski L, Maslinski W. DNA Methylation as a Future Therapeutic and Diagnostic Target in Rheumatoid Arthritis. Cells. 2019;8(9):953. 10.3390/cells8090953 PMC6770174 31443448

[36] Hedrich CM, Mäbert K, Rauen T, et al. DNA methylation in systemic lupus erythematosus. Epigenomics. 2017;9(4):505–525. 27885845 10.2217/epi-2016-0096 PMC6040049

[37] Saradna A, Do DC, Kumar S, et al. Macrophage polarization and allergic asthma. Transl Res. 2018;191:1–14. 29066321 10.1016/j.trsl.2017.09.002 PMC5776696

[38] Hattori N, Ushijima T. Analysis of DNA Methylation in Tissues Exposed to Inflammation. Methods Mol Biol. 2018;1725:185–199. 29322419 10.1007/978-1-4939-7568-6_16

[39] Scott M, De Sario A. DNA methylation changes in cystic fibrosis: cause or consequence? Clin Genet. 2020;98(1):3–9. 10.1111/cge.13731 32112395

[40] Somineni HK, Venkateswaran S, Kilaru V, et al. Blood-Derived DNA Methylation Signatures of Crohn’s Disease and Severity of Intestinal Inflammation. Gastroenterology. 2019;156(8):2254–2265.e2253. 30779925 10.1053/j.gastro.2019.01.270 PMC6529254

[41] Sun S, Barreiro LB. The epigenetically-encoded memory of the innate immune system. Curr Opin Immunol. 2020;65:7–13. 32220702 10.1016/j.coi.2020.02.002 PMC7529637

[42] Bray F, Ferlay J, Soerjomataram I, et al. Global cancer statistics 2018: GLOBOCAN estimates of incidence and mortality worldwide for 36 cancers in 185 countries. CA Cancer J Clin. 2018;68(6):394–424. 30207593 10.3322/caac.21492

[43] Hanahan D, Weinberg RA. Hallmarks of cancer: the next generation. Cell. 2011;144(5):646–674. 21376230 10.1016/j.cell.2011.02.013

[44] Chen L, Deng H, Cui H, et al. Inflammatory responses and inflammation-associated diseases in organs. Oncotarget. 2017;9(6):7204–7218. 29467962 10.18632/oncotarget.23208 PMC5805548

[45] Poetsch AR. The genomics of oxidative DNA damage, repair, and resulting mutagenesis. Comput Struct Biotechnol J. 2020;18:207–219. 31993111 10.1016/j.csbj.2019.12.013 PMC6974700

[46] Bouchard MJ, Navas-Martin S. Hepatitis B and C virus hepatocarcinogenesis: lessons learned and future challenges. Cancer Lett. 2011;305(2):123–143. 21168955 10.1016/j.canlet.2010.11.014 PMC3071446

[47] Hattori N, Ushijima T. Epigenetic impact of infection on carcinogenesis: mechanisms and applications. Genome Med. 2016;8(1). DOI:10.1186/s13073-016-0267-2 PMC4731931 26823082

[48] Mitra A, MacIntyre DA, Marchesi JR, et al. The vaginal microbiota, human papillomavirus infection and cervical intraepithelial neoplasia: what do we know and where are we going next? Microbiome. 2016;4(1):58. 27802830 10.1186/s40168-016-0203-0 PMC5088670

[49] Maeda M, Moro H, Ushijima T. Mechanisms for the induction of gastric cancer by Helicobacter pylori infection: aberrant DNA methylation pathway. Gastric Cancer. 2017;20(Suppl 1):8–15. 27718135 10.1007/s10120-016-0650-0

[50] Takeshima H, Ushijima T. Accumulation of genetic and epigenetic alterations in normal cells and cancer risk. NPJ Precis Oncol. 2019;3(1):7. 30854468 10.1038/s41698-019-0079-0 PMC6403339

[51] Eyvazi S, Vostakolaei MA, Dilmaghani A, et al. The oncogenic roles of bacterial infections in development of cancer. Microb Pathog. 2020;141:104019. 32006638 10.1016/j.micpath.2020.104019

[52] Nakagawa T, Matsusaka K, Misawa K, et al. Stratification of HPV-associated and HPV-negative oropharyngeal squamous cell carcinomas based on DNA methylation epigenotypes. Int J Cancer. 2020;146(9):2460–2474. 31997344 10.1002/ijc.32890

[53] Mur LA, Huws SA, Cameron SJ, et al. Lung cancer: a new frontier for microbiome research and clinical translation. Ecancermedicalscience. 2018;12:866. 30263057 10.3332/ecancer.2018.866 PMC6145518

[54] Dong J, Ma Q. Integration of inflammation, fibrosis, and cancer induced by carbon nanotubes. Nanotoxicology. 2019;13(9):1244–1274. 31537143 10.1080/17435390.2019.1651920 PMC6803058

[55] Husebø GR, Nielsen R, Hardie J, et al. Risk factors for lung cancer in COPD - results from the Bergen COPD cohort study. Respir Med. 2019;152:81–88. 31128615 10.1016/j.rmed.2019.04.019

[56] Hata A, Nakajima T, Matsusaka K, et al. A low DNA methylation epigenotype in lung squamous cell carcinoma and its association with idiopathic pulmonary fibrosis and poorer prognosis. Int J Cancer. 2020;146(2):388–399. 31241180 10.1002/ijc.32532

[57] Parris BA, O’Farrell HE, Fong KM, et al. Chronic obstructive pulmonary disease (COPD) and lung cancer: common pathways for pathogenesis. J Thorac Dis. 2019;11(Suppl 17):S2155–s2172. 31737343 10.21037/jtd.2019.10.54 PMC6831920

[58] Neglia JP, FitzSimmons SC, Maisonneuve P, et al. The risk of cancer among patients with cystic fibrosis. Cystic Fibrosis and Cancer Study Group. N Engl J Med. 1995;332(8):494–499. 7830730 10.1056/NEJM199502233320803

[59] Zhang Z, Wang J, He J, et al. Genetic variants in MUC4 gene are associated with lung cancer risk in a Chinese population. PLoS ONE. 2013;8(10):e77723. 24204934 10.1371/journal.pone.0077723 PMC3804582

[60] Dong Y, Zhou L, Zhao D, et al. MUC5AC enhances tumor heterogeneity in lung adenocarcinoma with mucin production and is associated with poor prognosis. Jpn J Clin Oncol. 2020;50(6):701–711. 32083303 10.1093/jjco/hyaa016

[61] Albino D, Longoni N, Curti L, et al. ESE3/EHF controls epithelial cell differentiation and its loss leads to prostate tumors with mesenchymal and stem-like features. Cancer Res. 2012;72(11):2889–2900. 22505649 10.1158/0008-5472.CAN-12-0212

[62] Jäger B, Klatt D, Plappert L, et al. CXCR4/MIF axis amplifies tumor growth and epithelial-mesenchymal interaction in non-small cell lung cancer. Cell Signal. 2020;73:109672. 32428553 10.1016/j.cellsig.2020.109672

[63] O’Leary K, Shia A, Schmid P. Epigenetic Regulation of EMT in Non-Small Cell Lung Cancer. Curr Cancer Drug Targets. 2018;18(1):89–96. 28176646 10.2174/1568009617666170203162556

[64] Warzecha CC, Carstens RP. Complex changes in alternative pre-mRNA splicing play a central role in the epithelial-to-mesenchymal transition (EMT). Semin Cancer Biol. 2012;22(5–6):417–427. 22548723 10.1016/j.semcancer.2012.04.003 PMC3413750

[65] Wright FA, Strug LJ, Doshi VK, et al. Genome-wide association and linkage identify modifier loci of lung disease severity in cystic fibrosis at 11p13 and 20q13.2. Nat Genet. 2011;43(6):539–546. 21602797 10.1038/ng.838 PMC3296486

[66] He Q, Wang L, Zhang J, et al. CYP2E1 and GSTM1 gene polymorphisms, environmental factors, and the susceptibility to lung cancer. J Clin Lab Anal. 2018;32(6):e22403. 29604112 10.1002/jcla.22403 PMC6817220

[67] Yu P, Kusuma JD, Suarez MAR, et al. Lung cancer susceptibility from GSTM1 deletion and air pollution with smoking status: a meta-prediction of worldwide populations. Oncotarget. 2018;9(57):31120–31132. 30123431 10.18632/oncotarget.25693 PMC6089566

[68] Grieshober L, Graw S, Barnett MJ, et al. AHRR methylation in heavy smokers: associations with smoking, lung cancer risk, and lung cancer mortality. BMC Cancer. 2020;20(1):905. 32962699 10.1186/s12885-020-07407-x PMC7510160

[69] Park KH, Lo Han SG, Whang YM, et al. Single nucleotide polymorphisms of the TGFB1 gene and lung cancer risk in a Korean population. Cancer Genet Cytogenet. 2006;169(1):39–44. 16875935 10.1016/j.cancergencyto.2006.03.012

[70] Risch A, Wikman H, Thiel S, et al. Glutathione-S-transferase M1, M3, T1 and P1 polymorphisms and susceptibility to non-small-cell lung cancer subtypes and hamartomas. Pharmacogenetics. 2001;11(9):757–764. 11740339 10.1097/00008571-200112000-00003

[71] Timofeeva MN, Hung RJ, Rafnar T, et al. Influence of common genetic variation on lung cancer risk: meta-analysis of 14 900 cases and 29 485 controls. Hum Mol Genet. 2012;21(22):4980–4995. 22899653 10.1093/hmg/dds334 PMC3607485

[72] Assenov Y, Müller F, Lutsik P, et al. Comprehensive Analysis of DNA Methylation Data with RnBeads. Nat Methods. 2014;11(11):1138–1140. 25262207 10.1038/nmeth.3115 PMC4216143

[73] Price ME, Cotton AM, Lam LL, et al. Additional annotation enhances potential for biologically-relevant analysis of the Illumina Infinium HumanMethylation450 BeadChip array. Epigenetics Chromatin. 2013;6(1):4. 23452981 10.1186/1756-8935-6-4 PMC3740789

[74] Kurdyukov S, Bullock M. DNA Methylation Analysis: choosing the Right Method. Biology (Basel). 2016;5(1):3. 10.3390/biology5010003 PMC4810160 26751487

[75] Reinius LE, Acevedo N, Joerink M, et al. Differential DNA Methylation in Purified Human Blood Cells: implications for Cell Lineage and Studies on Disease Susceptibility. PLoS ONE. 2012;7(7):e41361. 22848472 10.1371/journal.pone.0041361 PMC3405143

[76] Díez-Villanueva A, Mallona I, Peinado MA. Wanderer, an interactive viewer to explore DNA methylation and gene expression data in human cancer. Epigenetics Chromatin. 2015;8(1):22. 26113876 10.1186/s13072-015-0014-8 PMC4480445

[77] Ernst J, Kellis M. Discovery and characterization of chromatin states for systematic annotation of the human genome. Nat Biotechnol. 2010;28(8):817–825. 20657582 10.1038/nbt.1662 PMC2919626

[78] Ernst J, Kheradpour P, Mikkelsen TS, et al. Mapping and analysis of chromatin state dynamics in nine human cell types. Nature. 2011;473(7345):43–49. 21441907 10.1038/nature09906 PMC3088773

[79] Euskirchen GM, Rozowsky JS, Wei CL, et al. Mapping of transcription factor binding regions in mammalian cells by ChIP: comparison of array- and sequencing-based technologies. Genome Res. 2007;17(6):898–909. 17568005 10.1101/gr.5583007 PMC1891348

[80] Hudson ME, Snyder M. High-throughput methods of regulatory element discovery. Biotechniques. 2006;41(6):673, 675, 677 passim. 10.2144/000112322 17191608

[81] Grana O, Lopez-Fernandez H, Fdez-Riverola F, et al. Bicycle: a bioinformatics pipeline to analyze bisulfite sequencing data. Bioinformatics. 2018;34(8):1414–1415. 29211825 10.1093/bioinformatics/btx778

[82] Houseman EA, Accomando WP, Koestler DC, et al. DNA methylation arrays as surrogate measures of cell mixture distribution. BMC Bioinformatics. 2012;13(1):86. 22568884 10.1186/1471-2105-13-86 PMC3532182

[83] Hu H, Li B, Duan S. The Alteration of Subtelomeric DNA Methylation in Aging-Related Diseases. Front Genet. 2019;9:697. 30687384 10.3389/fgene.2018.00697 PMC6333653

[84] Elborn JS. Cystic fibrosis. Lancet. 2016;388(10059):2519–2531. 27140670 10.1016/S0140-6736(16)00576-6

[85] Bergman Y, Cedar H. DNA methylation dynamics in health and disease. Nat Struct Mol Biol. 2013;20(3):274–281. 23463312 10.1038/nsmb.2518

[86] Kaminsky ZA, Tang T, Wang S-C, et al. DNA methylation profiles in monozygotic and dizygotic twins. Nat Genet. 2009;41(2):240–245. 19151718 10.1038/ng.286

[87] Van Baak TE, Coarfa C, Dugué P-A, et al. Epigenetic supersimilarity of monozygotic twin pairs. Genome Biol. 2018;19(1):2. 29310692 10.1186/s13059-017-1374-0 PMC5759268

[88] Downey DG, Bell SC, Elborn JS. Neutrophils in cystic fibrosis. Thorax. 2009;64(1):81–88. 19103874 10.1136/thx.2007.082388

[89] Holliday R, Pugh J. DNA modification mechanisms and gene activity during development. Science. 1975;187(4173):226–232. 1111098

[90] Levy H, Kalish LA, Huntington I, et al. Inflammatory markers of lung disease in adult patients with cystic fibrosis. Pediatr Pulmonol. 2007;42(3):256–262. 17245735 10.1002/ppul.20563 PMC4469989

[91] Terry MB, Delgado-Cruzata L, Vin-Raviv N, et al. DNA methylation in white blood cells: association with risk factors in epidemiologic studies. Epigenetics. 2011;6(7):828–837. 21636973 10.4161/epi.6.7.16500 PMC3154425

[92] Guida F, Sandanger TM, Castagné R, et al. Dynamics of smoking-induced genome-wide methylation changes with time since smoking cessation. Hum Mol Genet. 2015;24(8):2349–2359. 25556184 10.1093/hmg/ddu751 PMC4380075

[93] Teschendorff AE, Yang Z, Wong A, et al. Correlation of Smoking-Associated DNA Methylation Changes in Buccal Cells With DNA Methylation Changes in Epithelial Cancer. JAMA Oncol. 2015;1(4):476–485. 26181258 10.1001/jamaoncol.2015.1053

[94] Zhou G, Yang L, Gray A, et al. The role of desmosomes in carcinogenesis. Onco Targets Ther. 2017;10:4059–4063. 28860814 10.2147/OTT.S136367 PMC5565390

[95] Bibikova M, Barnes B, Tsan C, et al. High density DNA methylation array with single CpG site resolution. Genomics. 2011;98(4):288–295. 21839163 10.1016/j.ygeno.2011.07.007

[96] Doi A, Park IH, Wen B, et al. Differential methylation of tissue- and cancer-specific CpG island shores distinguishes human induced pluripotent stem cells, embryonic stem cells and fibroblasts. Nat Genet. 2009;41(12):1350–1353. 19881528 10.1038/ng.471 PMC2958040

[97] Irizarry RA, Ladd-Acosta C, Wen B, et al. The human colon cancer methylome shows similar hypo- and hypermethylation at conserved tissue-specific CpG island shores. Nat Genet. 2009;41(2):178–186. 19151715 10.1038/ng.298 PMC2729128

[98] Ziller MJ, Gu H, Müller F, et al. Charting a dynamic DNA methylation landscape of the human genome. Nature. 2013;500(7463):477–481. 23925113 10.1038/nature12433 PMC3821869

[99] Neri F, Rapelli S, Krepelova A, et al. Intragenic DNA methylation prevents spurious transcription initiation. Nature. 2017;543(7643):72–77. 28225755 10.1038/nature21373

[100] Yang X, Han H, De Carvalho DD, et al. Gene body methylation can alter gene expression and is a therapeutic target in cancer. Cancer Cell. 2014;26(4):577–590. 25263941 10.1016/j.ccr.2014.07.028 PMC4224113

[101] Arechederra M, Daian F, Yim A, et al. Hypermethylation of gene body CpG islands predicts high dosage of functional oncogenes in liver cancer. Nat Commun. 2018;9(1):3164. 30089774 10.1038/s41467-018-05550-5 PMC6082886

[102] Gu J, Stevens M, Xing X, et al. Mapping of Variable DNA Methylation Across Multiple Cell Types Defines a Dynamic Regulatory Landscape of the Human Genome. G3 (Bethesda). 2016;6(4):973–986. 26888867 10.1534/g3.115.025437 PMC4825665

[103] Arzate-Mejía RG, Recillas-Targa F, Corces VG. Developing in 3D: the role of CTCF in cell differentiation. Development. 2018;145(6):dev137729. 29567640 10.1242/dev.137729 PMC5897592

[104] Herold M, Bartkuhn M, Renkawitz R. CTCF: insights into insulator function during development. Development. 2012;139(6):1045–1057. 22354838 10.1242/dev.065268

[105] Ruiz-Velasco M, Kumar M, Lai MC, et al. CTCF-Mediated Chromatin Loops between Promoter and Gene Body Regulate Alternative Splicing across Individuals. Cell Syst. 2017;5(6):628–637.e626. 29199022 10.1016/j.cels.2017.10.018

[106] Ambrosi C, Manzo M, Baubec T. Dynamics and Context-Dependent Roles of DNA Methylation. J Mol Biol. 2017;429(10):1459–1475. 28214512 10.1016/j.jmb.2017.02.008

[107] Lévêque M, Le Trionnaire S, Del Porto P, et al. The impact of impaired macrophage functions in cystic fibrosis disease progression. J Cyst Fibros. 2017;16(4):443–453. 27856165 10.1016/j.jcf.2016.10.011

[108] Barnes PJ. Cellular and molecular mechanisms of asthma and COPD. Clin Sci. 2017;131(13):1541–1558. 10.1042/CS20160487 28659395

[109] Houghton AM. Mechanistic links between COPD and lung cancer. Nat Rev Cancer. 2013;13(4):233–245. 23467302 10.1038/nrc3477

[110] Shields PG, Berman M, Brasky TM, et al. A Review of Pulmonary Toxicity of Electronic Cigarettes in the Context of Smoking: a Focus on Inflammation. Cancer Epidemiol Biomarkers Prev. 2017;26(8):1175–1191. 28642230 10.1158/1055-9965.EPI-17-0358 PMC5614602

[111] Siegel RL, Miller KD, Jemal A. Cancer statistics, 2019. CA Cancer J Clin. 2019;69(1):7–34. 30620402 10.3322/caac.21551

[112] Strzelak A, Ratajczak A, Adamiec A, et al. Tobacco Smoke Induces and Alters Immune Responses in the Lung Triggering Inflammation, Allergy, Asthma and Other Lung Diseases: a Mechanistic Review. Int J Environ Res Public Health. 2018;15(5):1033. 10.3390/ijerph15051033 PMC5982072 29883409

[113] Chaparro C, Keshavjee S. Lung transplantation for cystic fibrosis: an update. Expert Rev Respir Med. 2016;10(12):1269–1280. 27842444 10.1080/17476348.2016.1261016

[114] Ranganathan SC, Hall GL, Sly PD, et al., & Australian Respiratory Early Surveillance Team for Cystic, F. Early Lung Disease in Infants and Preschool Children with Cystic Fibrosis. What Have We Learned and What Should We Do about It? Am J Respir Crit Care Med. 2017;195(12):1567–1575. . 10.1164/rccm.201606-1107CI PMC6850725 27911585

[115] Li Y, Sun Z, Wu Y, et al. Cystic fibrosis transmembrane conductance regulator gene mutation and lung cancer risk. Lung Cancer. 2010;70(1):14–21. 20116881 10.1016/j.lungcan.2010.01.005 PMC2895007

[116] Bronsveld I, Mekus F, Bijman J, et al. Chloride conductance and genetic background modulate the cystic fibrosis phenotype of ΔF508 homozygous twins and siblings. J Clin Investig. 2001;108(11):1705–1715. 11733566 10.1172/JCI12108 PMC200980

[117] MacGregor AJ, Snieder H, Schork NJ, et al. Twins: novel uses to study complex traits and genetic diseases. Trends Genet. 2000;16(3):131–134. 10689354 10.1016/s0168-9525(99)01946-0

[118] Gentilini D, Garagnani P, Pisoni S, et al. Stochastic epigenetic mutations (DNA methylation) increase exponentially in human aging and correlate with X chromosome inactivation skewing in females. Aging (Albany NY). 2015;7(8):568–578. 26342808 10.18632/aging.100792 PMC4586102

[119] Jones PA, Baylin SB. The epigenomics of cancer. Cell. 2007;128(4):683–692. 17320506 10.1016/j.cell.2007.01.029 PMC3894624

[120] Bell JT, Tsai PC, Yang TP, et al. Epigenome-wide scans identify differentially methylated regions for age and age-related phenotypes in a healthy ageing population. PLoS Genet. 2012;8(4):e1002629. 22532803 10.1371/journal.pgen.1002629 PMC3330116

[121] Boks MP, Derks EM, Weisenberger DJ, et al. The relationship of DNA methylation with age, gender and genotype in twins and healthy controls. PLoS ONE. 2009;4(8):e6767. 19774229 10.1371/journal.pone.0006767 PMC2747671

[122] Hannon E, Knox O, Sugden K, et al. Characterizing genetic and environmental influences on variable DNA methylation using monozygotic and dizygotic twins. PLoS Genet. 2018;14(8):e1007544. 30091980 10.1371/journal.pgen.1007544 PMC6084815

[123] Horvath S. DNA methylation age of human tissues and cell types. Genome Biol. 2013;14(10):R115. 24138928 10.1186/gb-2013-14-10-r115 PMC4015143

[124] Liu J, Morgan M, Hutchison K, et al. A study of the influence of sex on genome wide methylation. PLoS ONE. 2010;5(4):e10028. 20386599 10.1371/journal.pone.0010028 PMC2850313

[125] Teschendorff AE, Menon U, Gentry-Maharaj A, et al. Age-dependent DNA methylation of genes that are suppressed in stem cells is a hallmark of cancer. Genome Res. 2010;20(4):440–446. 20219944 10.1101/gr.103606.109 PMC2847747

[126] Yet I, Tsai P-C, Castillo-Fernandez JE, et al. Genetic and environmental impacts on DNA methylation levels in twins. Epigenomics. 2016;8(1):105–117. 26678685 10.2217/epi.15.90

[127] Fraga MF, Ballestar E, Paz MF, et al. Epigenetic differences arise during the lifetime of monozygotic twins. Proc Natl Acad Sci U S A. 2005;102(30):10604–10609. 16009939 10.1073/pnas.0500398102 PMC1174919

[128] Talens RP, Christensen K, Putter H, et al. Epigenetic variation during the adult lifespan: cross-sectional and longitudinal data on monozygotic twin pairs. Aging Cell. 2012;11(4):694–703. 22621408 10.1111/j.1474-9726.2012.00835.x PMC3399918

[129] Artis D, Spits H. The biology of innate lymphoid cells. Nature. 2015;517(7534):293–301. 25592534 10.1038/nature14189

[130] Brown TA, Lee JW, Holian A, et al. Alterations in DNA methylation corresponding with lung inflammation and as a biomarker for disease development after MWCNT exposure. Nanotoxicology. 2016;10(4):453–461. 26375518 10.3109/17435390.2015.1078852 PMC4779417

[131] Ehrlich M. DNA hypermethylation in disease: mechanisms and clinical relevance. Epigenetics. 2019;14(12):1141–1163. 31284823 10.1080/15592294.2019.1638701 PMC6791695

[132] Issa J-P. Aging and epigenetic drift: a vicious cycle. J Clin Invest. 2014;124(1):24–29. 24382386 10.1172/JCI69735 PMC3871228

[133] Nunes SP, Diniz F, Moreira-Barbosa C, et al. Subtyping Lung Cancer Using DNA Methylation in Liquid Biopsies. J Clin Med. 2019;8(9):1500. 10.3390/jcm8091500 PMC6780554 31546933

[134] Gunasekara CJ, Scott CA, Laritsky E, et al. A genomic atlas of systemic interindividual epigenetic variation in humans. Genome Biol. 2019;20(1):105. 31155008 10.1186/s13059-019-1708-1 PMC6545702

[135] Bonné S, Gilbert B, Hatzfeld M, et al. Defining desmosomal plakophilin-3 interactions. J Cell Biol. 2003;161(2):403–416. 12707304 10.1083/jcb.200303036 PMC2172904

[136] Dusek RL, Attardi LD. Desmosomes: new perpetrators in tumour suppression. Nat Rev Cancer. 2011;11(5):317–323. 21508970 10.1038/nrc3051 PMC3799918

[137] Hatzfeld M, Wolf A, Keil R. Plakophilins in desmosomal adhesion and signaling. Cell Commun Adhes. 2014;21(1):25–42. 24460199 10.3109/15419061.2013.876017

[138] Hofmann I, Casella M, Schnölzer M, et al. Identification of the junctional plaque protein plakophilin 3 in cytoplasmic particles containing RNA-binding proteins and the recruitment of plakophilins 1 and 3 to stress granules. Mol Biol Cell. 2006;17(3):1388–1398. 16407409 10.1091/mbc.E05-08-0708 PMC1382326

[139] Schmidt A, Langbein L, Prätzel S, et al. Plakophilin 3 – a novel cell-type-specific desmosomal plaque protein. Differentiation. 1999;64(5):291–306. 10374265 10.1046/j.1432-0436.1999.6450291.x

[140] Adam D, Roux-Delrieu J, Luczka E, et al. Cystic fibrosis airway epithelium remodelling: involvement of inflammation. J Pathol. 2015;235(3):408–419. 25348090 10.1002/path.4471

[141] Besnard V, Dagher R, Madjer T, et al. Identification of periplakin as a major regulator of lung injury and repair in mice. JCI Insight. 2018;3(5). DOI:10.1172/jci.insight.90163 PMC5922284 29515024

[142] Müller L, Rietscher K, Keil R, et al. Plakophilin 3 phosphorylation by ribosomal S6 kinases supports desmosome assembly. J Cell Sci. 2020;133:8. 10.1242/jcs.238295 32122945

[143] Breuninger S, Reidenbach S, Sauer CG, et al. Desmosomal plakophilins in the prostate and prostatic adenocarcinomas: implications for diagnosis and tumor progression. Am J Pathol. 2010;176(5):2509–2519. 20348237 10.2353/ajpath.2010.090737 PMC2861115

[144] Demirag GG, Sullu Y, Yucel I. Expression of Plakophilins (PKP1, PKP2, and PKP3) in breast cancers. Med Oncol. 2012;29(3):1518–1522. 21947748 10.1007/s12032-011-0071-1

[145] Takahashi H, Nakatsuji H, Takahashi M, et al. Up-regulation of plakophilin-2 and Down-regulation of plakophilin-3 are correlated with invasiveness in bladder cancer. Urology. 2012;79(1):240.e241–248. 10.1016/j.urology.2011.08.049 22119253

[146] Valladares-Ayerbes M, Diaz-Prado S, Reboredo M, et al. Evaluation of plakophilin-3 mRNA as a biomarker for detection of circulating tumor cells in gastrointestinal cancer patients. Cancer Epidemiol Biomarkers Prev. 2010;19(6):1432–1440. 20501752 10.1158/1055-9965.EPI-10-0123

[147] Furukawa C, Daigo Y, Ishikawa N, et al. Plakophilin 3 oncogene as prognostic marker and therapeutic target for lung cancer. Cancer Res. 2005;65(16):7102–7110. 16103059 10.1158/0008-5472.CAN-04-1877

[148] Sklyarova T, Bonne S, D’Hooge P, et al. Plakophilin-3-deficient mice develop hair coat abnormalities and are prone to cutaneous inflammation. J Invest Dermatol. 2008;128(6):1375–1385. 18079750 10.1038/sj.jid.5701189

[149] Sklyarova T, van Hengel J, Van Wonterghem E, et al. Hematopoietic plakophilin-3 regulates acute tissue-specific and systemic inflammation in mice. Eur J Immunol. 2015;45(10):2898–2910. 26173741 10.1002/eji.201445440

[150] Chae YK, Choi WM, Bae WH, et al. Overexpression of adhesion molecules and barrier molecules is associated with differential infiltration of immune cells in non-small cell lung cancer. Sci Rep. 2018;8(1):1023. 29348685 10.1038/s41598-018-19454-3 PMC5773521

[151] Bruscia EM, Bonfield TL. Innate and Adaptive Immunity in Cystic Fibrosis. Clin Chest Med. 2016;37(1):17–29. 26857765 10.1016/j.ccm.2015.11.010

[152] Borghese B, Barbaux S, Mondon F, et al. Research resource: genome-wide profiling of methylated promoters in endometriosis reveals a subtelomeric location of hypermethylation. Mol Endocrinol. 2010;24(9):1872–1885. 20685852 10.1210/me.2010-0160 PMC5417403

[153] Dyson MT, Roqueiro D, Monsivais D, et al. Genome-wide DNA methylation analysis predicts an epigenetic switch for GATA factor expression in endometriosis. PLoS Genet. 2014;10(3):e1004158. 24603652 10.1371/journal.pgen.1004158 PMC3945170

[154] Toiyama Y, Okugawa Y, Kondo S, et al. Comprehensive analysis identifying aberrant DNA methylation in rectal mucosa from ulcerative colitis patients with neoplasia. Oncotarget. 2018;9(69):33149–33159. 30237858 10.18632/oncotarget.26032 PMC6145694

[155] Li L, Wei J, Li S, et al. The deubiquitinase USP13 stabilizes the anti-inflammatory receptor IL-1R8/Sigirr to suppress lung inflammation. EBioMedicine. 2019;45:553–562. 31204278 10.1016/j.ebiom.2019.06.011 PMC6642080

[156] Horne DJ, Randhawa AK, Chau TT, et al. Common polymorphisms in the PKP3-SIGIRR-TMEM16J gene region are associated with susceptibility to tuberculosis. J Infect Dis. 2012;205(4):586–594. 22223854 10.1093/infdis/jir785 PMC3266131

[157] Blanchard AC, Waters VJ. Microbiology of Cystic Fibrosis Airway Disease. Semin Respir Crit Care Med. 2019;40(6):727–736. 31887768 10.1055/s-0039-1698464 PMC7117079

[158] Françoise A, Héry-Arnaud G. The Microbiome in Cystic Fibrosis Pulmonary Disease. Genes (Basel). 2020;11(5):536. 10.3390/genes11050536 PMC7288443 32403302

