# Supplementary figures and images for: Disease-related blood-based differential methylation in cystic fibrosis and its representation in lung cancer revealed a regulatory locus in PKP3 in lung epithelial cells
Source: Epigenetics. 2021 Aug 20;17(8):837–60. doi: 10.1080/15592294.2021.1959976 (PMC9423854; doi:10.1080/15592294.2021.1959976)

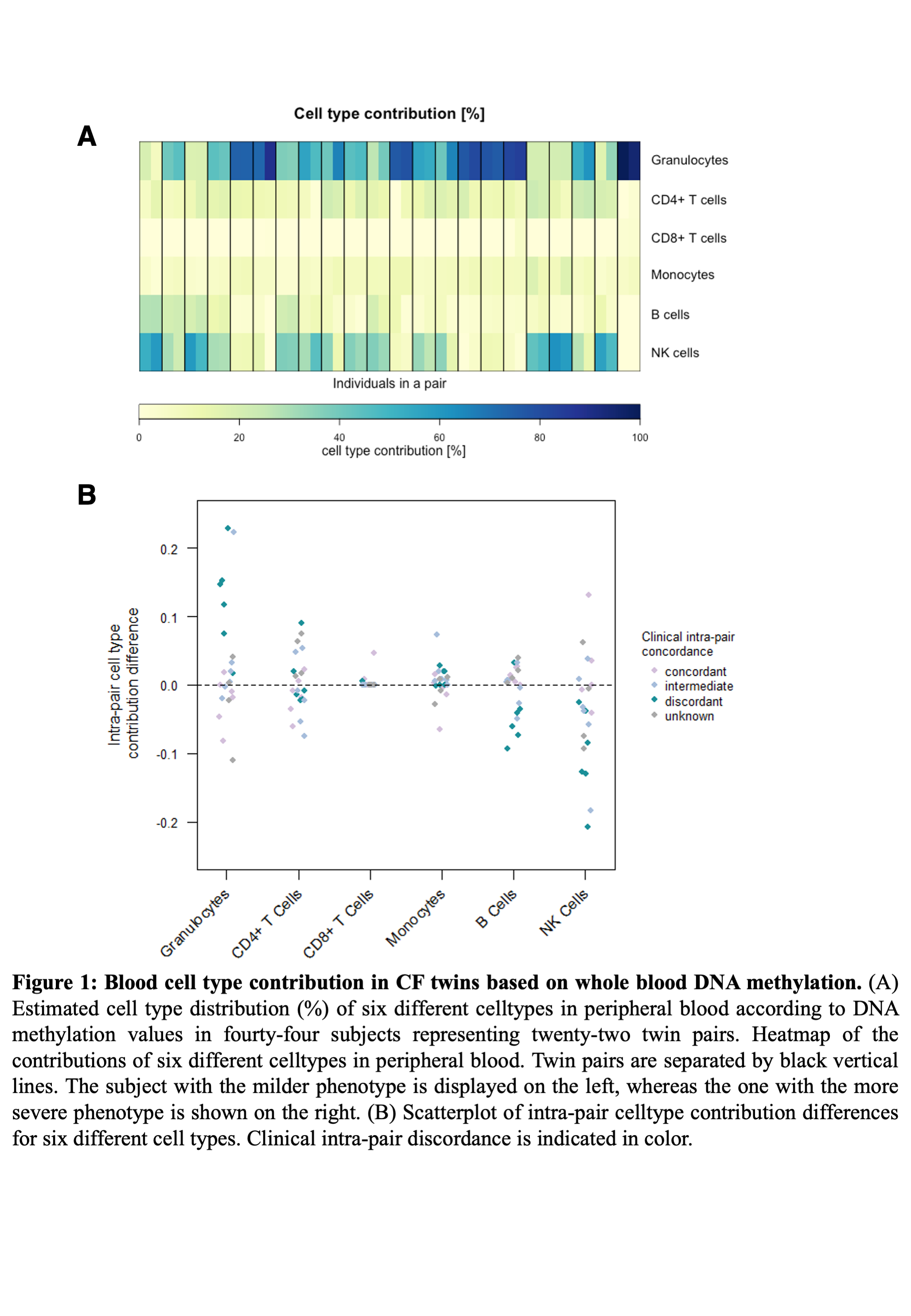

Supplement: Supplemental Material [file KEPI_A_1959976_SM5507.zip › supplementary/SupFig1.tiff]

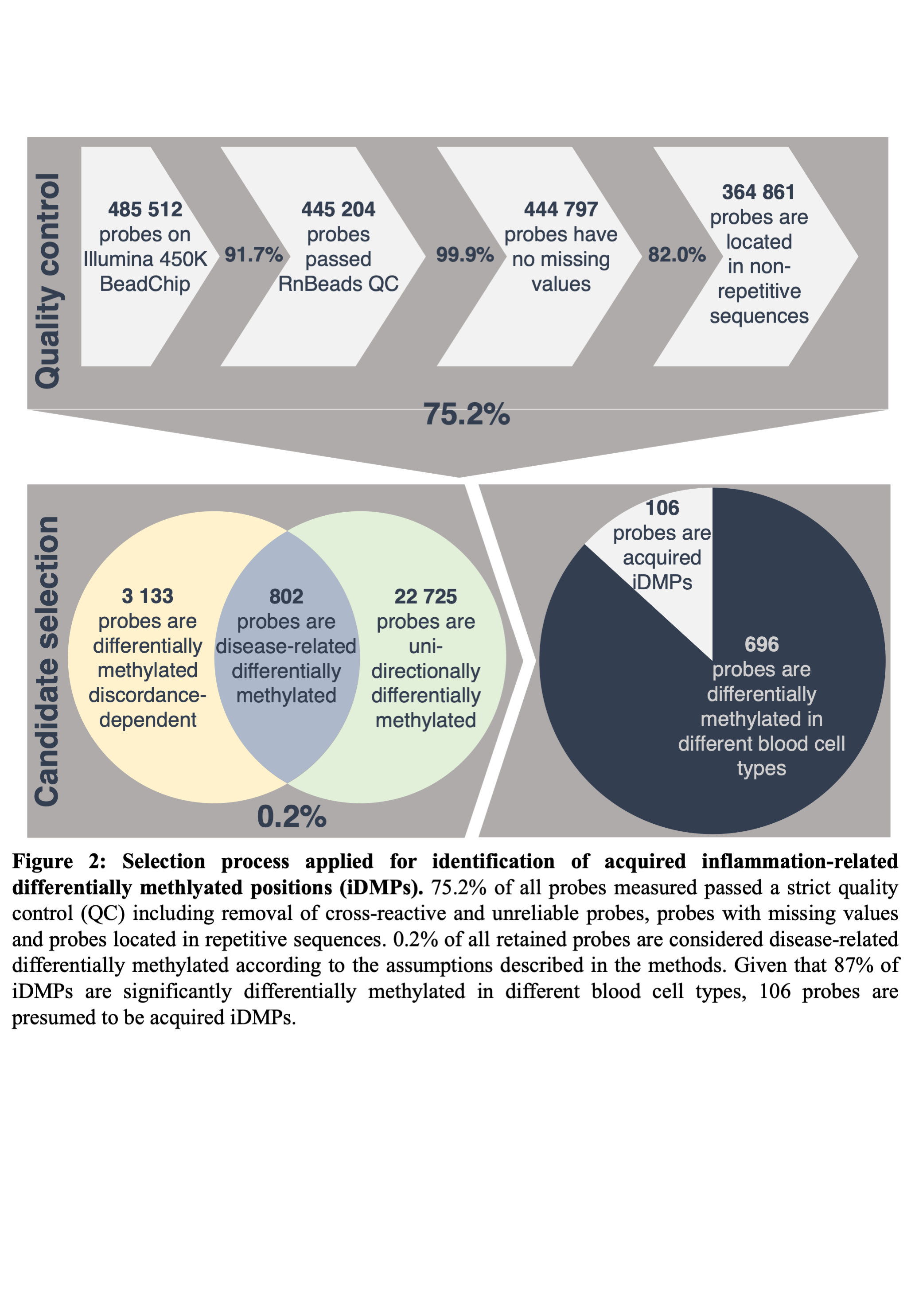

Supplement: Supplemental Material [file KEPI_A_1959976_SM5507.zip › supplementary/SupFig2.tiff]

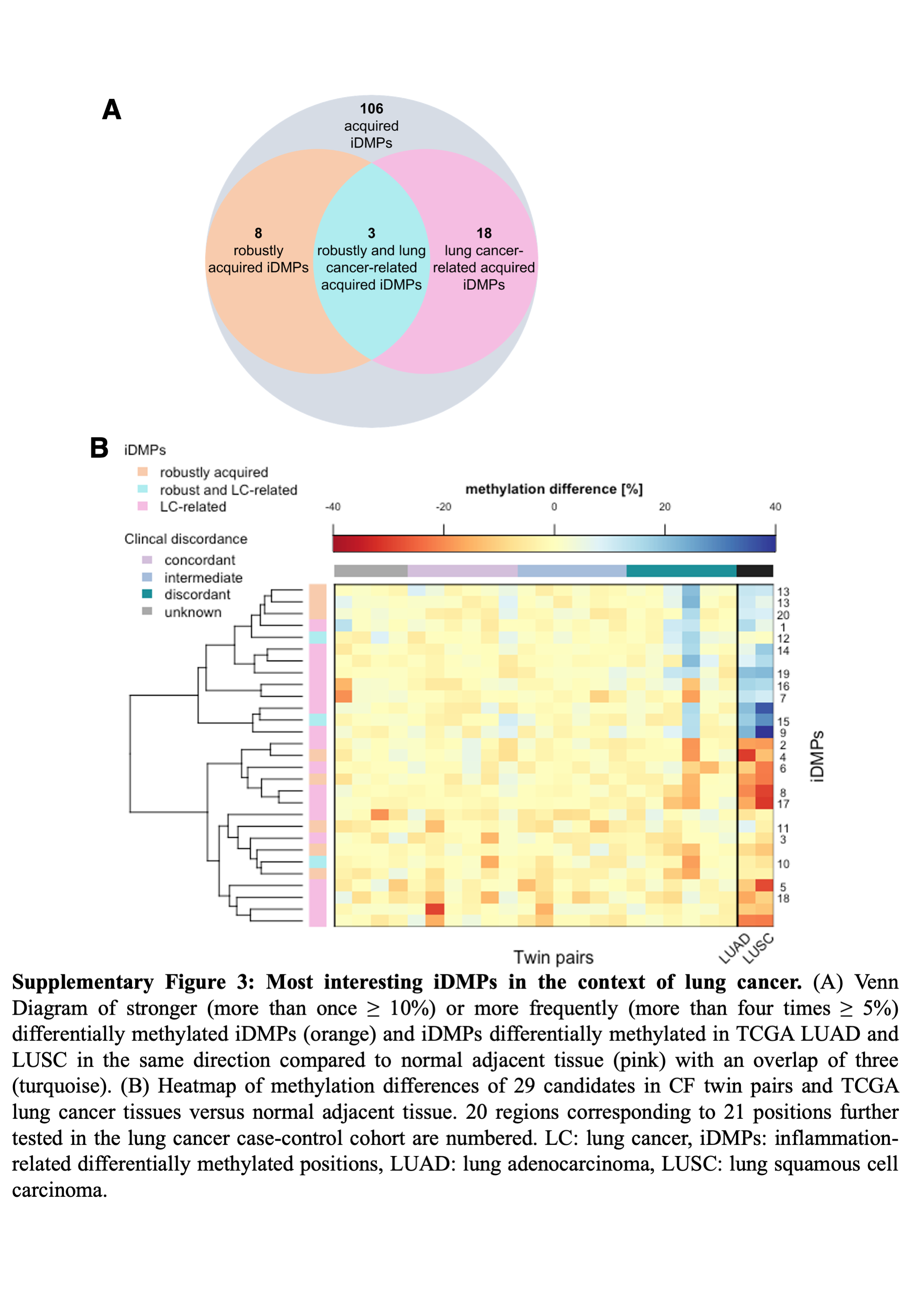

Supplement: Supplemental Material [file KEPI_A_1959976_SM5507.zip › supplementary/SupFig3.tiff]

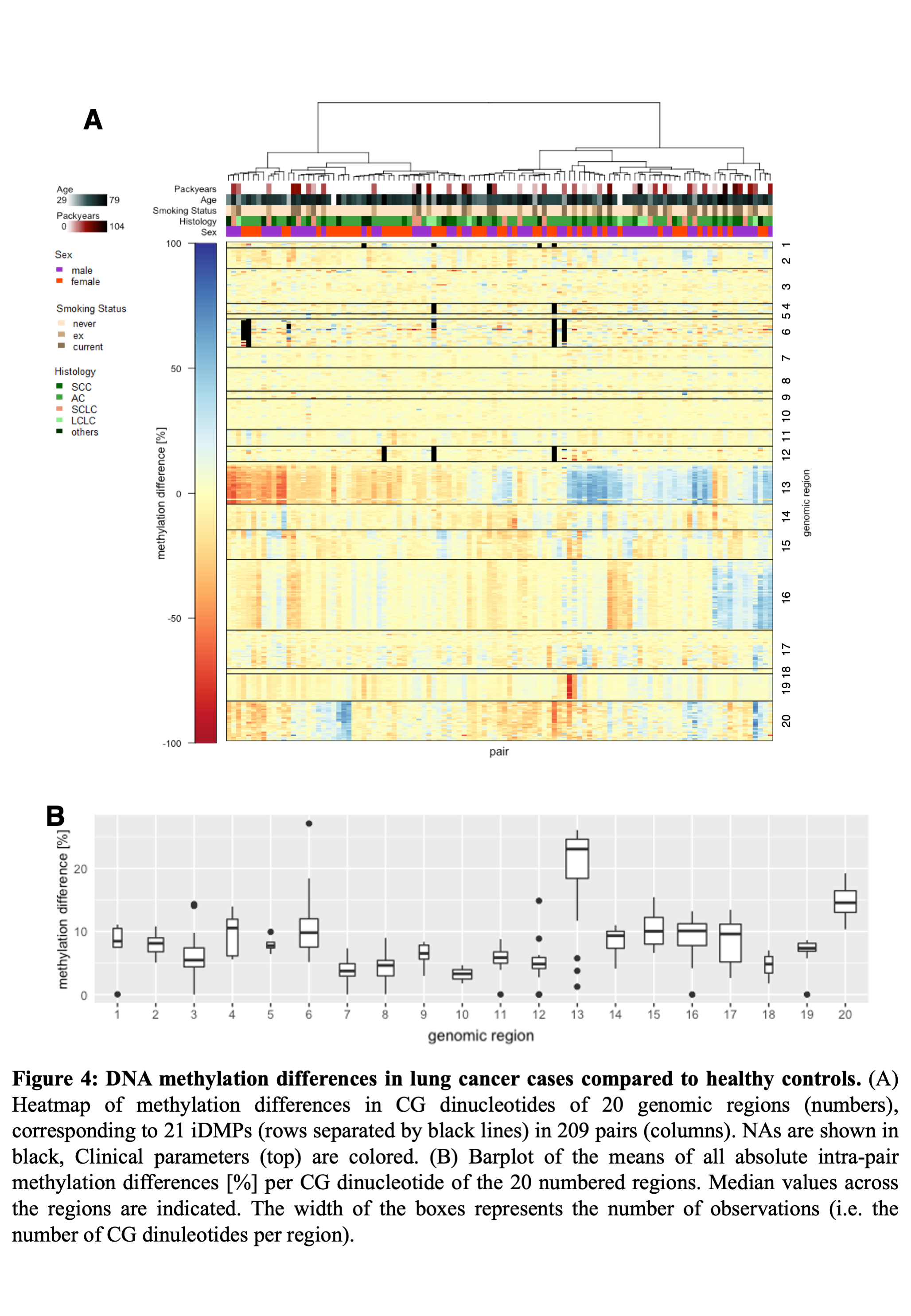

Supplement: Supplemental Material [file KEPI_A_1959976_SM5507.zip › supplementary/SupFig4.tiff]

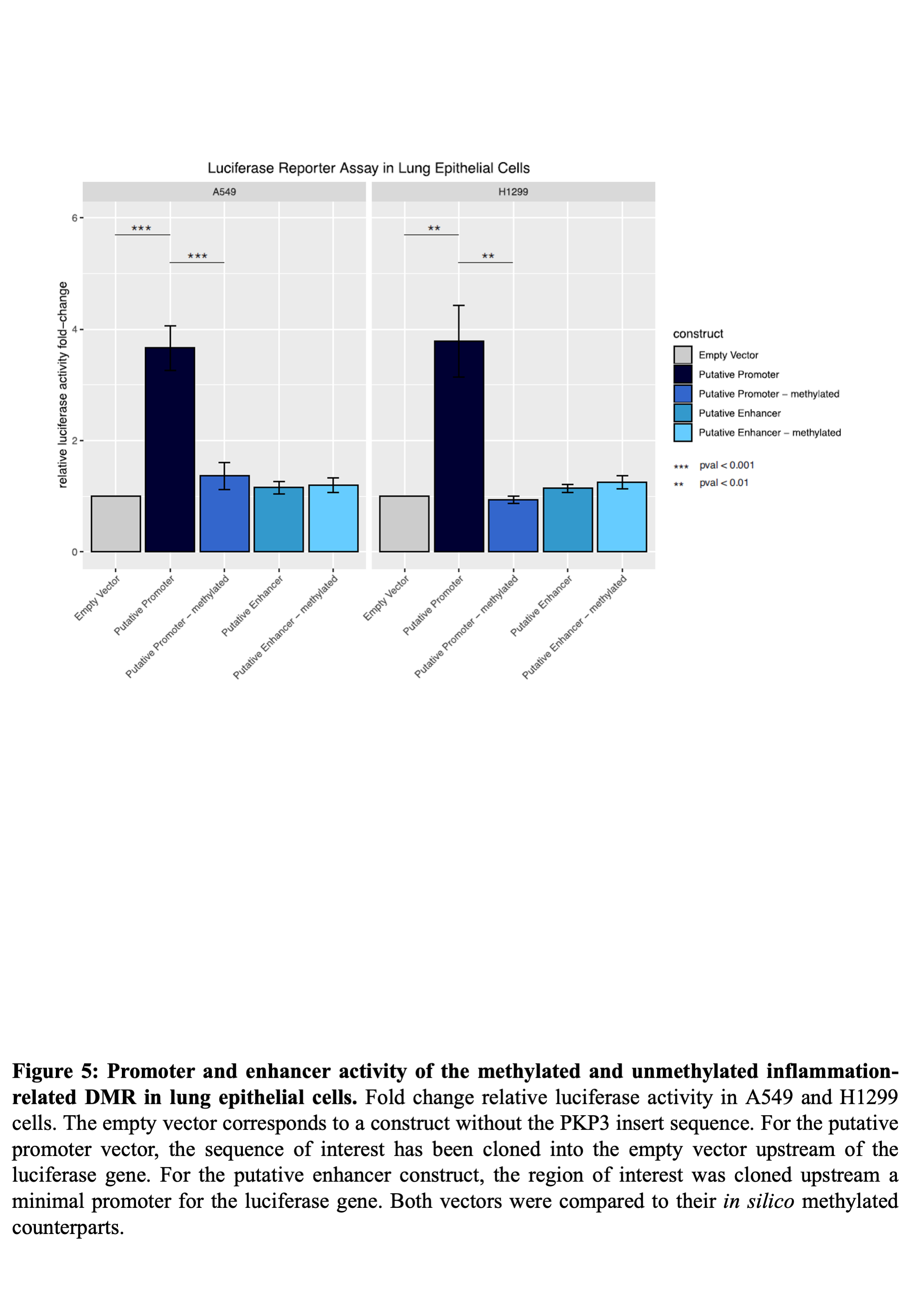

Supplement: Supplemental Material [file KEPI_A_1959976_SM5507.zip › supplementary/SupFig5.tiff]

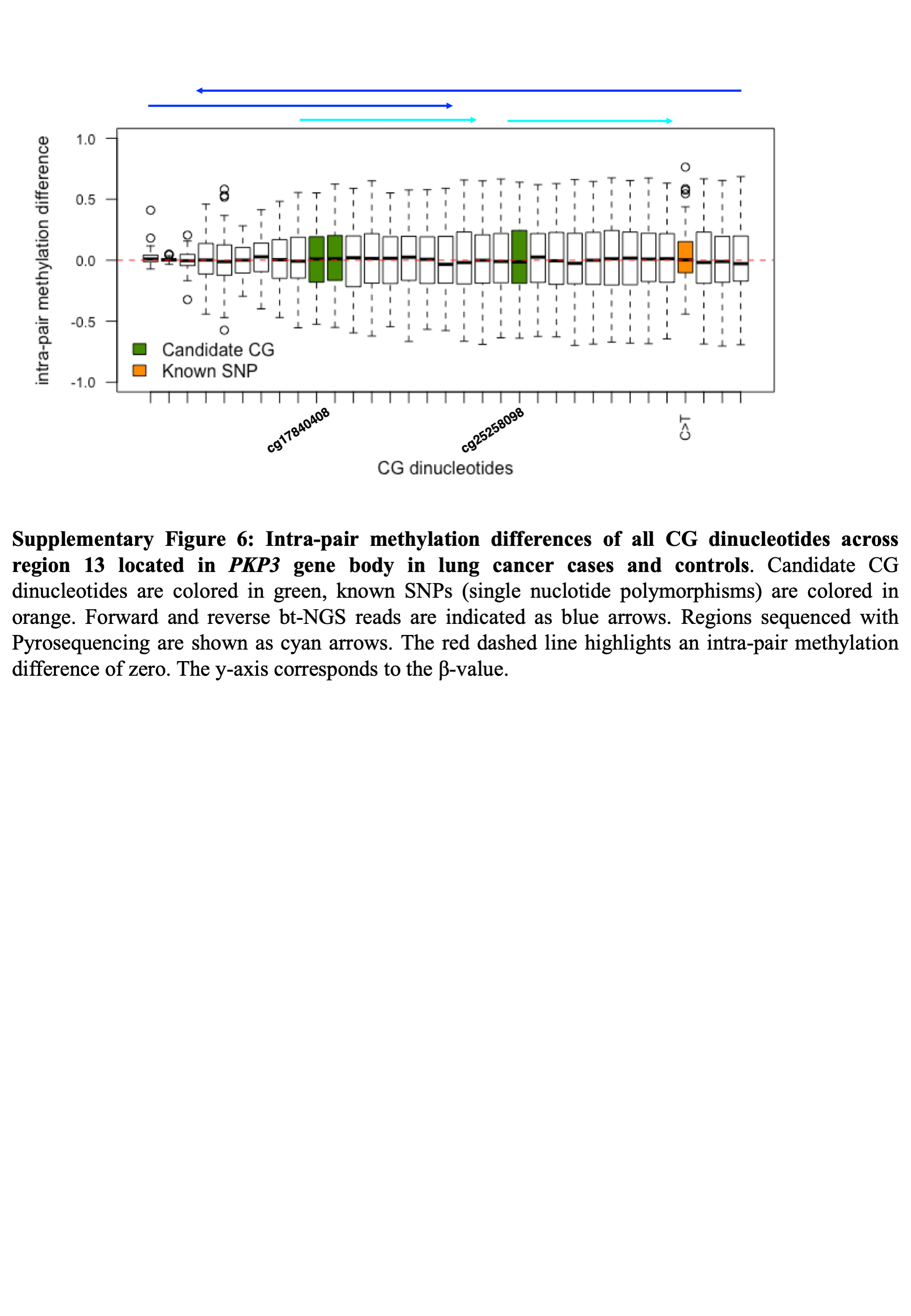

Supplement: Supplemental Material [file KEPI_A_1959976_SM5507.zip › supplementary/SupFig6.tiff]

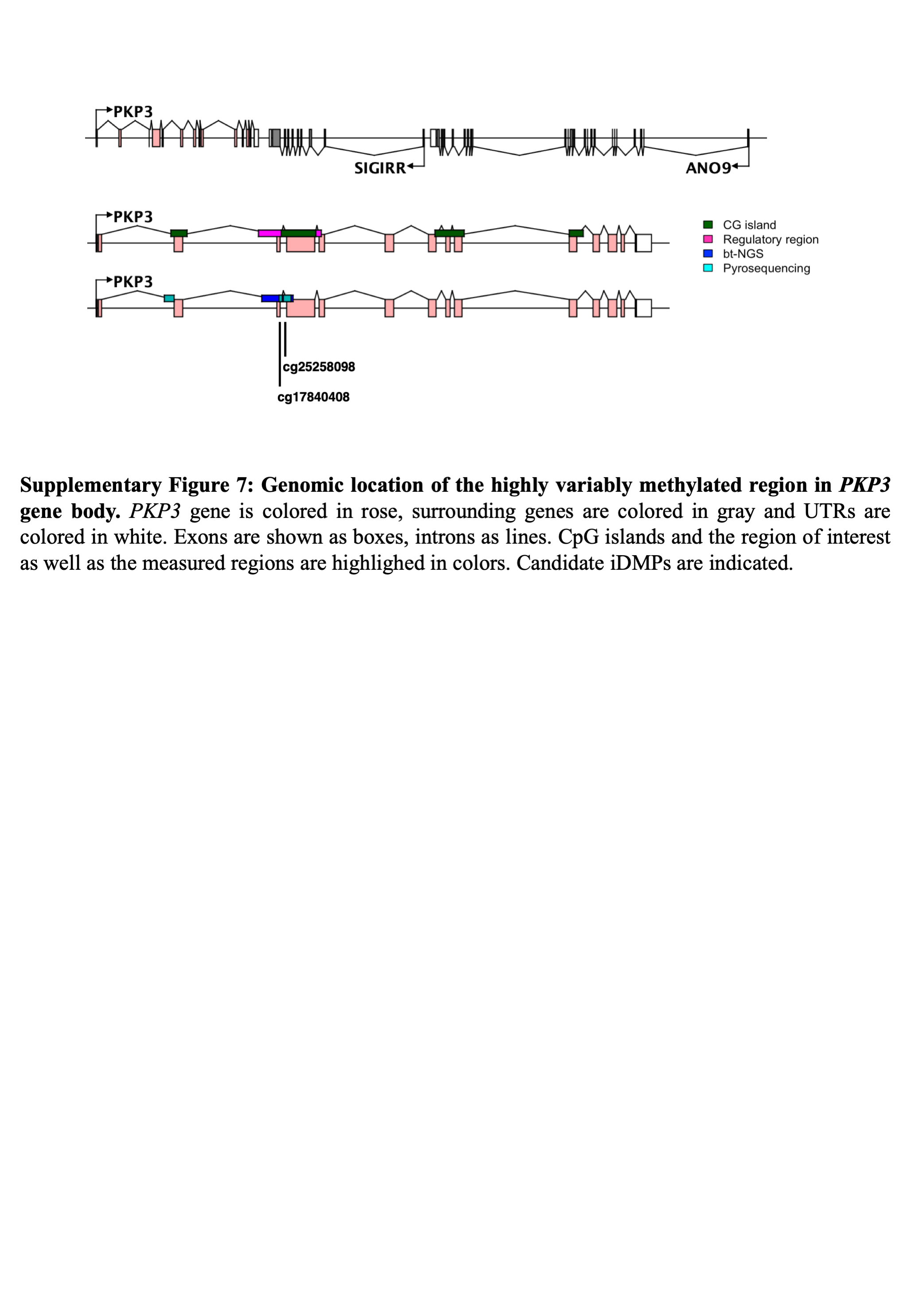

Supplement: Supplemental Material [file KEPI_A_1959976_SM5507.zip › supplementary/SupFig7.tiff]

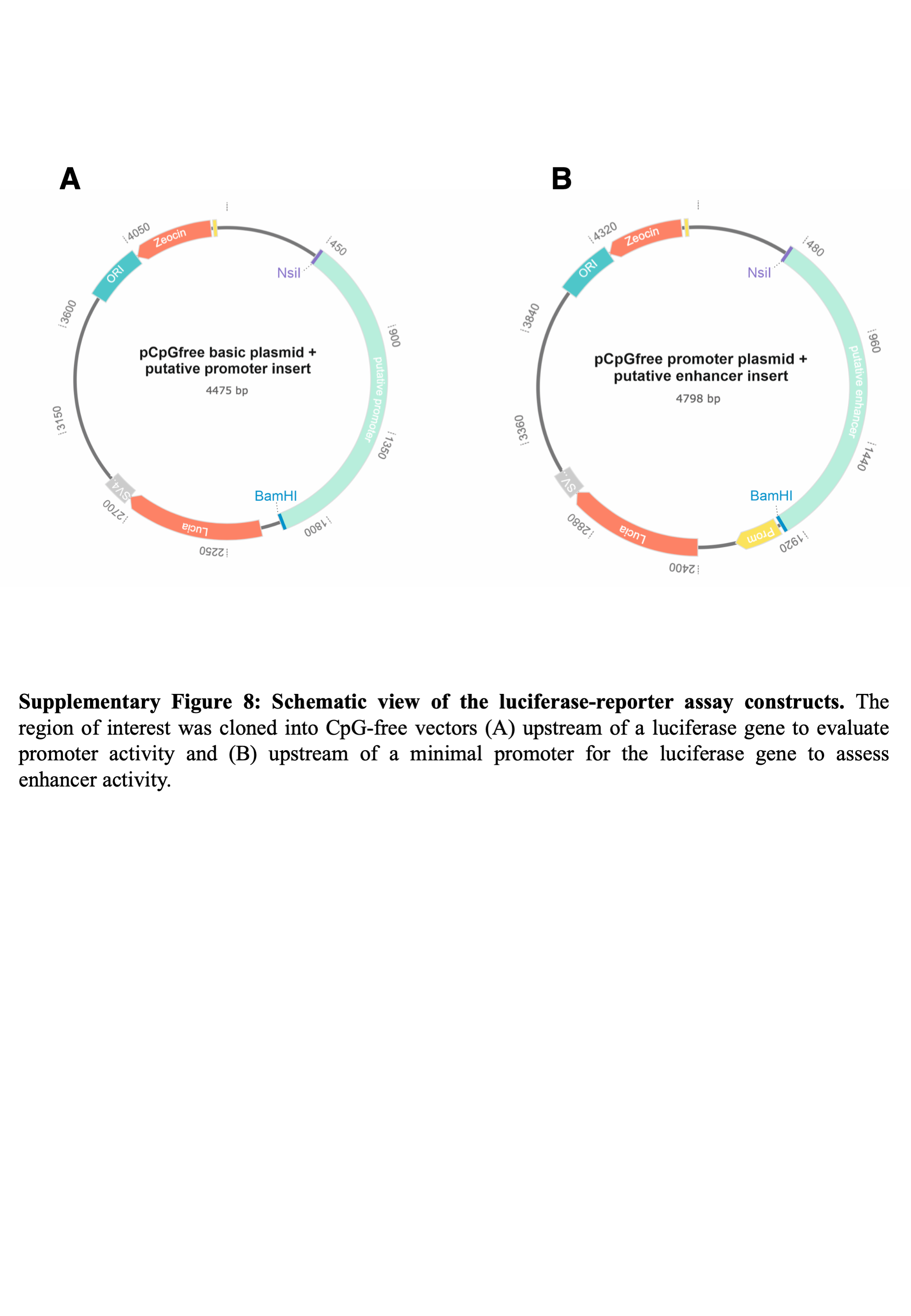

Supplement: Supplemental Material [file KEPI_A_1959976_SM5507.zip › supplementary/SupFig8.tiff]
